# The combination of supervised and unsupervised learning based risk stratification and phenotyping in pulmonary arterial hypertension—a long-term retrospective multicenter trial

**DOI:** 10.1186/s12890-023-02427-2

**Published:** 2023-04-25

**Authors:** Thomas Sonnweber, Piotr Tymoszuk, Regina Steringer-Mascherbauer, Elisabeth Sigmund, Stephanie Porod-Schneiderbauer, Lisa Kohlbacher, Igor Theurl, Irene Lang, Günter Weiss, Judith Löffler-Ragg

**Affiliations:** 1 Department of Internal Medicine II, Medical University of Innsbruck, Anichstraße 35, 6020 Innsbruck, Austria; 2 Data Analytics As a Service Tirol, Daas.Tirol, Innsbruck, Austria; 3 Department of Cardiology, Elisabethinenkrankenhaus, Linz, Austria; 4 Department of Cardiology, Medical University of Vienna, Vienna, Austria

**Keywords:** Pulmonary arterial hypertension, Risk assessment, Biomarkers, Mortality, Right-heart failure, Atypical pulmonary arterial hypertension

## Abstract

**Background:**

Accurate risk stratification in pulmonary arterial hypertension (PAH), a devastating cardiopulmonary disease, is essential to guide successful therapy. Machine learning may improve risk management and harness clinical variability in PAH.

**Methods:**

We conducted a long-term retrospective observational study (median follow-up: 67 months) including 183 PAH patients from three Austrian PAH expert centers. Clinical, cardiopulmonary function, laboratory, imaging, and hemodynamic parameters were assessed. Cox proportional hazard Elastic Net and partitioning around medoid clustering were applied to establish a multi-parameter PAH mortality risk signature and investigate PAH phenotypes.

**Results:**

Seven parameters identified by Elastic Net modeling, namely age, six-minute walking distance, red blood cell distribution width, cardiac index, pulmonary vascular resistance, N-terminal pro-brain natriuretic peptide and right atrial area, constituted a highly predictive mortality risk signature (training cohort: concordance index = 0.82 [95%CI: 0.75 – 0.89], test cohort: 0.77 [0.66 – 0.88]). The Elastic Net signature demonstrated superior prognostic accuracy as compared with five established risk scores. The signature factors defined two clusters of PAH patients with distinct risk profiles. The high-risk/poor prognosis cluster was characterized by advanced age at diagnosis, poor cardiac output, increased red cell distribution width, higher pulmonary vascular resistance, and a poor six-minute walking test performance.

**Conclusion:**

Supervised and unsupervised learning algorithms such as Elastic Net regression and medoid clustering are powerful tools for automated mortality risk prediction and clinical phenotyping in PAH.

**Supplementary Information:**

The online version contains supplementary material available at 10.1186/s12890-023-02427-2.

## Background

Pulmonary arterial hypertension (PAH) is a rare disease with a detrimental long-term outcome [1–4]⁠. Hemodynamically, PAH is defined by precapillary pulmonary hypertension with an elevated mean pulmonary arterial pressure (mPAP) > 20 mmHg, a pulmonary arterial wedge pressure ≤ 15 mmHg, and an elevated pulmonary vascular resistance (PVR) > 2 Wood units in right heart catheterization (RHC) at rest [5, 6]⁠.

Despite the progress in therapy, which dramatically improved clinical outcome, complete disease control is often not achieved and long-term survival of PAH patients remains low [7, 8]⁠. PAH treatment decisions are based on individualized risk assessment at diagnosis and follow-up [5, 9]⁠. Still, optimal PAH risk management has not been achieved yet, and available PAH risk stratification tools are highly heterogeneous, differ in the choice, number and weighting of used parameters, and the risk class definition [1, 8–13]. It has been recognized that the current definition of PAH includes heterogeneous patient phenotypes which differ in treatment responses and clinical outcomes [14, 15]⁠. Following this observation, typical and atypical forms of PAH have been described, whereas the latter term refers to a late-onset PAH in patients with multiple comorbidities as compared to typical PAH, which is found in younger patients with few comorbidities [14]⁠. Such phenotyping of PAH is essential to further improve individualized risk stratification and treatment decisions, which is addressed by numerous recent reports [5]. Current research on PAH risk assessment focuses primarily on novel risk parameters including imaging, functional, genetic, and proteomic features [16, 17]⁠. Still, the implementation of such new risk markers in clinical practice is often hindered by a lack of availability, standardization, and increasing costs. By contrast, there are several more easily accessible features, such as age and sex, PAH etiology, serological parameters, pulmonary function tests, cardiac and pulmonary imaging, electrocardiography, exercise testing, and RHC measures which have been shown to predict mortality in PAH but are inconsistently used in currently available PAH risk scores [18–20]⁠. Thus, we hypothesized that a machine learning approach employing e. g. Elastic Net survival modeling and clustering based on a broad set of easily accessible demographic and clinical parameters available at PAH diagnosis may facilitate risk stratification and identification of clinically relevant subsets of PAH patients.

## Methods

### Ethics

All participants gave written informed consent to participate. The study data were stored and analyzed in anonymized form. The study was approved by the ethics committees of the Medical University of Innsbruck (approval numbers: AM2544, 239/4.12 and 273/5.7), the Johannes Keppler University of Linz (AN2017-0,009,369/4.15) and the Medical University of Vienna (EKV516/2011) and conducted in accordance with the Declaration of Helsinki and European data policy.

### Study population and design

**Fig. 1:**
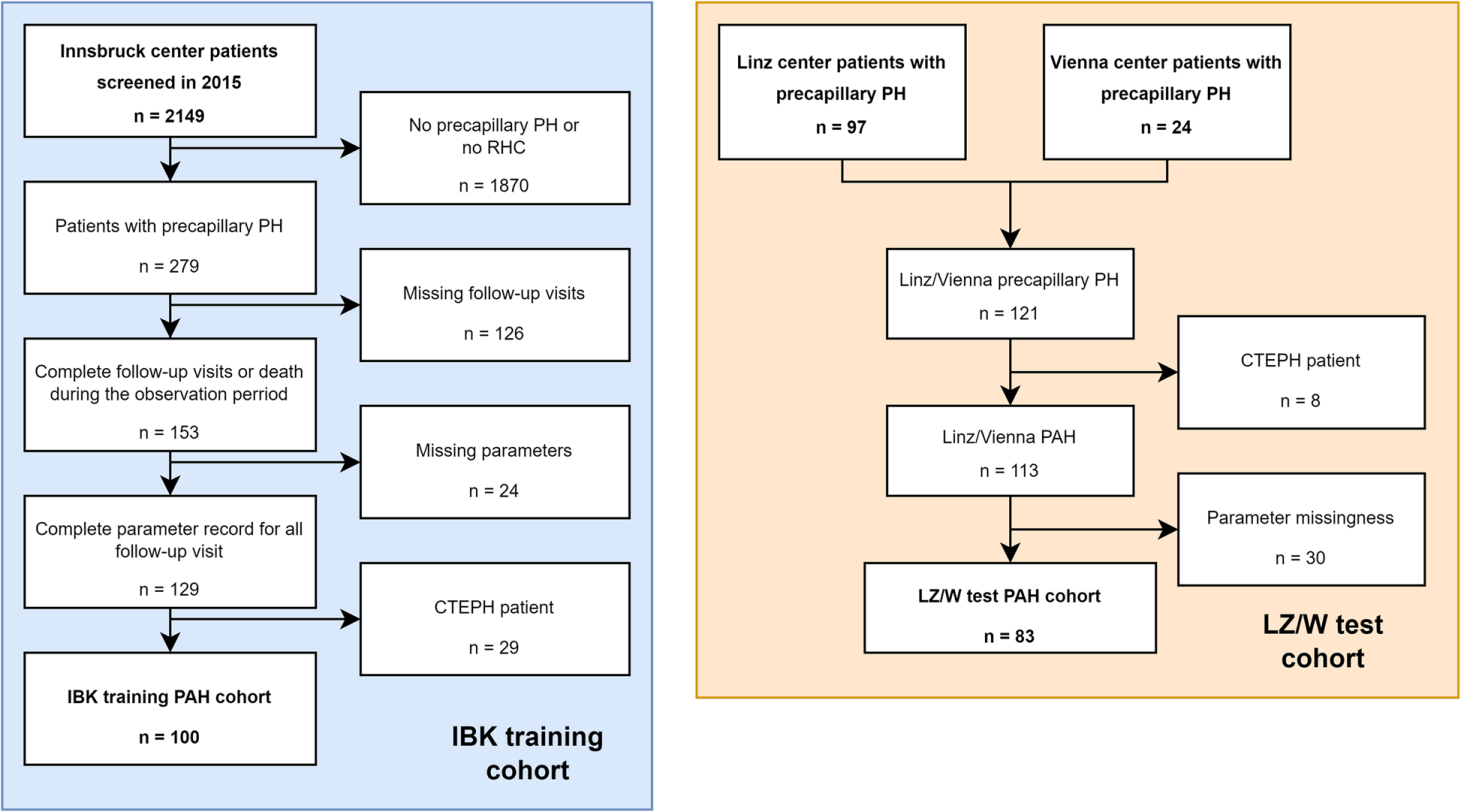
Flow diagram of the study analysis inclusion process. PH: pulmonary hypertension; RHC: right heart catheterization; CTEPH: chronic thromboembolic pulmonary hypertension

We herein present a retrospective multicenter observational two-cohort study. Data of 183 PAH patients were analyzed. The inclusion criterion was PAH (WHO etiology group I) defined by ESC/ERS (European Society of Cardiology/European Respiratory Society) guidelines and confirmed by RHC. The exclusion criterion was incompatibility with PAH diagnosis, e. g. post-capillary pulmonary hypertension or pulmonary hypertension group II, III, IV, or V. The participants were grouped in (1) the training cohort recruited at the Medical University of Innsbruck, Austria, (IBK, *N* = 100) and the (2) test cohort recruited at the Elisabethinen Hospital Linz, Austria, and the Medical University of Vienna, Austria (LZ/W, *N* = 83) (Fig. 1).

### Study procedures and variables

Demographic and performance variables, blood biomarkers, heart echocardiography, and RHC parameters were determined during the standard PAH diagnosis procedure. Capillary blood gas analysis was performed by puncture of the hyper-perfused earlobe (induced by Finalgon® application [Sanofi-Aventis, Germany]). The glomerular filtration rate (GFR) was calculated by the MDRD GFR equation, renal insufficiency was defined as GFR < 60 ml/min/1.73 m^2^. Anemia was defined by a hemoglobin concentration < 120 g/L for women and < 130 g/L for men. Impaired oxygenation was defined as peripheral hemoglobin oxygen saturation (SO_2_) < 95%. The list of analyzed variables with the stratification scheme is presented in Supplementary Table S1.

### Mortality risk-assessment tools

The mortality risk at diagnosis was assessed by abbreviated versions of the ESC/ERS risk assessment tool: the three- and four parameter French Pulmonary Hypertension Registry scores (FPHR 3p and 4p), the Prospective Registry of Newly Initiated Therapies for Pulmonary Hypertension (COMPERA) tool, the Swedish PAH register (SPAHR) model, and the modified Risk Assessment Score of PAH (mRASP) [8, 13, 21, 22]⁠.

### Statistical analysis

Statistical analysis was performed with R version 4.2.3 (R Foundation for Statistical Computing).

In descriptive statistic, numeric variables are presented as medians with interquartile ranges (IQR). Categorical variables are presented as percentages and counts for each category. Prior to modeling and clustering, numeric variables were normalized and median centered. Both first and second order terms for numeric variables were included in models to account for non-linear relationship with survival.

Statistical significance was assessed by Mann–Whitney, χ2, log-rank and Spearman’s test for numeric variables, categorical parameters, survival and correlation, respectively. *P* values were adjusted for multiple testing within each analysis and cohort with the Benjamini–Hochberg method [23]⁠. Association of single factors (Supplementary Table S1) or established risk assessment tools (mRASP, SPAHR, COMPERA, FPHR) with overall survival was investigated by univariable Cox proportional hazard modeling [24]⁠. For multi-parameter survival modeling, the Elastic Net model [25, 26]⁠ was trained in the IBK cohort with the optimal lambda parameter obtained in 200-repetition tenfold cross-validation. Subsequently, the Elastic Net model linear predictor scores were calculated for the training IBK and test LZ/W cohort and their association with overall survival was assessed by univariable Cox modeling [24, 27, 28]⁠. Performance of survival models was measured by concordance index (C) [29]⁠, integrated Brier score (IBS) [30]⁠ and R2 explained variance statistics. Non-zero model coefficients in the Elastic Net model (hazard ratio ≠ 1) were deemed the ‘Elastic Net signature’. Clustering of the IBK cohort participants in respect to the Elastic Net signature variables was done with PAM (partition around medoids) algorithm and cosine distance measure [31, 32]⁠. The optimal cluster number was determined by the bend of the within-cluster sum of squares curve, the peak silhouette statistic [33]⁠, and the largest cluster assignment accuracy in tenfold cross-validation [34]⁠. Cluster assignment in the LZ/W collective was accomplished by the inverse distance-weighted 7-nearest neighbor classifier [35]⁠. Details of statistical analysis are provided in Supplementary Methods. The analysis pipeline is available at https://github.com/PiotrTymoszuk/PAH-biomarker.

## Results

### Characteristics of the study cohorts

The PAH study cohorts (total: *N* = 183, IBK: *N* = 100, LZ/W: *N* = 83, Fig. 1) encompassed subjects with idiopathic (*N* = 172, 94%), hereditary (*N* = 3, 1.6%) and connective tissue disease associated PAH (*N* = 8, 4.4%). The latter group consisted of patients with systemic sclerosis, systemic lupus erythematosus, Sjörgen syndrome and mixed connective tissue disease. The median age was 66 years [IQR: 53 – 71] in the IBK and 70 years [IQR: 54 – 74] in the LZ/W collective. The cohorts included predominately females (IBK: 64% and LZ/W: 66%) (Table 1). We found no significant differences in observation time (p = 0.30, Mann–Whitney test), overall survival (p = 0.51, log-rank test) and overall mortality rate (p = 0.35, χ2 test) between the IBK and LZ/W cohorts. Accordingly, the median overall survival defined as follow-up time between the diagnosis and the last visit or death was 70 months [IQR: 46 – 110] in the IBK and 63 months [IQR: 32 – 110] in the LZ/W cohort, and mortality during follow-up was 33% in the IBK and 24% in the LZ/W cohort (Table 1, Supplementary Tables S1 – S2).

**Table 1:** Characteristics of the Innsbruck (IBK) and Linz/Vienna (LZ/W) study cohorts. Numeric variables are presented as medians with interquartile ranges (IQR) and ranges. Categorical variables are presented as percentages and counts within the complete observation set

Variable^a^	IBK	LZ/W	Significance^b^	Effect size^b^
Participants, n	100	83		
Age, years	66 [IQR: 53—71] 19—84	70 [IQR: 54—74] 23—82	ns (*p* = 0.36)	*r* = 0.093
Sex	female: 64% (64) male: 36% (36)	female: 66% (55) male: 34% (28)	ns (*p* = 0.94)	V = 0.024
Anemia	19% (19)	17% (14)	ns (*p* = 0.94)	V = 0.028
Renal insufficiency	35% (35)	18% (15)	*p* = 0.043	V = 0.19
Percardial effusion	16% (16)	3.6% (3)	*p* = 0.04	V = 0.2
WHO class	I/II: 39% (39) III/IV: 61% (61)	I/II: 53% (44) III/IV: 47% (39)	ns (*p* = 0.17)	V = 0.14
SMWD, m	320 [IQR: 200—400] 50—610	350 [IQR: 270—440] 50—620	ns (*p* = 0.11)	*r* = 0.15
mPAP, mmHg	40 [IQR: 30—50] 26—120	39 [IQR: 31—49] 18—67	ns (*p* = 0.91)	*r* = 0.024
PVR, Wood	10 [IQR: 6.7—17] 3.3—43	5 [IQR: 3.5—7.8] 1.4—20	*p* < 0.001	*r* = 0.54
5-year mortality	21% (21)	13% (11)	ns (*p* = 0.38)	V = 0.1
OS, months	70 [IQR: 46—110] 2—230	63 [IQR: 32—110] 11—170	ns (*p* = 0.51)	

^a^WHO class: WHO functional class; SMWD: six-minute walking distance; mPAP: mean pulmonary arterial pressure; PVR: pulmonary vascular resistance; OS: overall survival

^b^Numeric variables: Mann–Whitney U test with r effect size statistic; categorical variables: χ^2^ test with Cramer V effect size statistic; survival: log-rank test

### Univariable survival modeling

Initially, we applied univariable Cox modeling to search for survival-associated factors among 19 demographic, performance, biochemical and cardiopulmonary parameters measured at diagnosis (Supplementary Table S1). By this approach, N-terminal pro-brain natriuretic peptide (NT-pro-BNP), pulmonary vascular resistance (PVR) and six-minute walking distance (SMWD) were identified as significant survival-associated factors in both the IBK and LZ/W cohorts. Additionally, age, mean corpuscular volume, right atrial area (RAA), renal insufficiency were linked to a worse prognosis and cardiac index (CI) was associated with better survival in the IBK collective. In the LZ/W cohort, male sex and III/IV WHO functional class were identified as unfavorable prognostic factors (Supplementary Figure S2, Supplementary Table S3).

### Development of a multi-parameter PAH risk signature with Elastic Net modeling

**Fig. 2:**
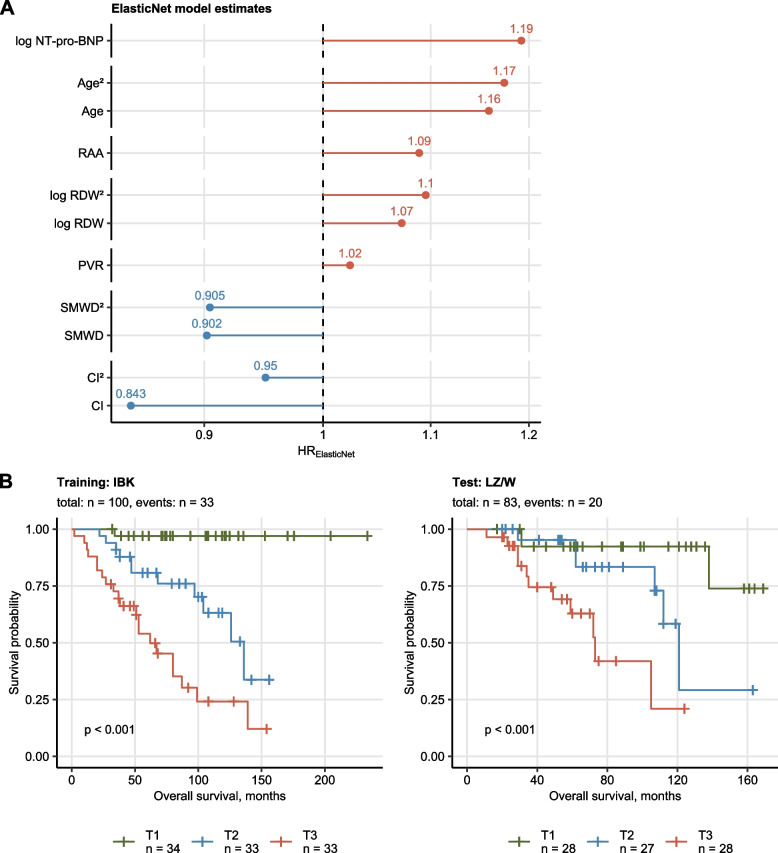
Multi-parameter modeling of PAH survival with Elastic Net Cox regression. The Elastic Net multi-parameter Cox regression model with the set of 19 (Supplementary Table S1) independent variables and overall survival as a response was developed in the training Innsbruck cohort. Numeric independent variables were median centered and their first and second order terms included in the model. Numbers of complete observations and mortality is indicated in B. **A** Non-zero Elastic Net model coefficients (Elastic Net signature) represented as hazard ratios (HR). Plot points are labeled with their HR values. **B** Association of overall survival with the Elastic Net model linear prediction score in the training IBK and test Linz/Vienna (LZ/W) cohort was assessed by Kaplan–Meier analysis. Significance of the survival differences in the study participants stratified by the linear predictor score tertiles (T1: 0—33, T2: 34—66, T3: 66—100 percentile) was determined by log-rank test adjusted for multiple testing with Benjamini–Hochberg method. *P* values are shown in the plots, numbers of complete observations and mortality are indicated in the plot captions

Multi-parameter modeling of overall survival was performed with Elastic Net Cox regression [25, 26]⁠. In the training IBK cohort, 7 out of 19 candidate independent variables, namely age, CI, NT-pro-BNP, PVR, RAA, RDW (red blood cell distribution width) and SMWD, contributed to risk prediction as reflected by hazard ratio ≠ 1 and constituted the ‘Elastic Net signature’. In particular, age at diagnosis (linear: hazard ratio [HR] = 1.16, quadratic term: HR = 1.17) and NT-pro-BNP (linear term: HR = 1.19) were the strongest  predictors of an unfavorable clinical course, whereas CI (linear: HR = 0.843, quadratic term: 0.95) and SMWD (linear: 0.902, quadratic term: 0.905) were linked to better overall survival (Fig. 2A).

The Elastic Net signature displayed good survival prediction accuracy [29, 30] in the training IBK (C = 0.82, R2 = 0.65, IBS = 0.098) and the test LZ/W collective (C = 0.77, R2 = 0.52, IBS = 0.11) (Supplementary Table S4). Accordingly, the linear predictor tertiles of the Elastic Net signature were associated with a low, intermediate and high risk of overall mortality in the Kaplan–Meier analysis in both cohorts indicating proper model calibration (Fig. 2B).

### Prediction of overall survival by the Elastic Net signature and established PAH risk assessment tools

The Elastic Net signature values correlated significantly with risk class assignment by mRASP, COMPERA and SPAHR, and a number of risk factors in the FPHR models. However, the strength of correlation of the Elastic Net signature with other tools (Spearman’s ρ, IBK: 0.57 – 0.7, LZ/W: 0.7 – 0.79) tended to be lower than the correlation of the established risk tools with each other (IBK: 0.61 – 0.9, LZ/W: 0.68 – 0.9) (Supplementary Figure S2). This suggests that the Elastic Net signature may provide better risk estimates for PAH individuals with inadequate survival prediction by other tools.

**Fig. 3:**
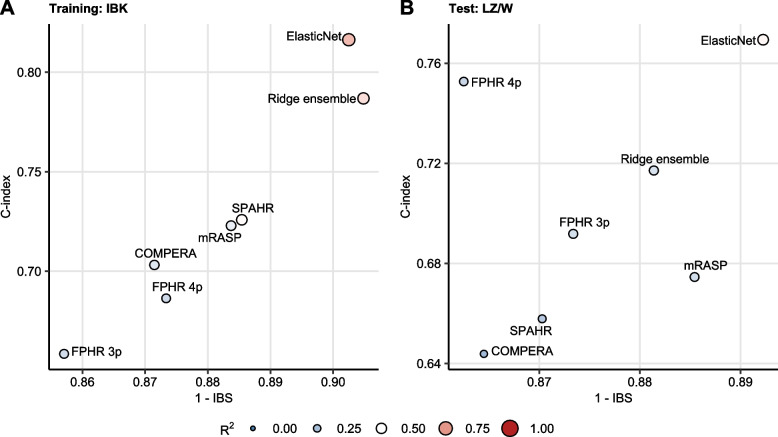
Performance of PAH risk assessment tools. The Elastic Net signature was developed as presented in Fig. 2. The ensemble of the established risk assessment tools (FPHR 3p: French Pulmonary Hypertension Registry 3 parameter score, FPHR 4p: French Pulmonary Hypertension Registry 4 parameter score, COMPERA: Comparative, Prospective Registry of Newly Initiated Therapies for Pulmonary Hypertension score, mRASP: modified Risk Assessment Score of PAH) was developed by Ridge Cox regression as presented in Supplementary Figure S3. Predictive performance of the Elastic Net signature, ensemble and single PAH risk scores at predicting overall survival was assessed by concordance index (C-index) and integrated Brier score (IBS). C-indexes and IBS for the risk assessment tools in the Innsbruck (IBK) and Linz/Vienna (LZ/W) cohorts are displayed in scatter plots, point size and color codes for ${R}^{2}$

To test for that, we compared predictive performance of the newly developed Elastic Net signature with the established risk assessment tools and an ensemble of the established risk scales developed with the Ridge Cox technique [25, 26](Supplementary Figure S4). In such comparison, the Elastic Net signature displayed a better prediction accuracy gauged by C-index, IBS and R2 than the best performing comparator risk scales. Furthermore, although the performance of the ensemble model and the Elastic Net signature in the IBK cohort was comparable, the ensemble of established risk scales performed substantially worse in the LZ/W collective (Fig. 3, Supplementary Table S4).

### Identification of PAH risk phenotypes by clustering

**Fig. 4:**
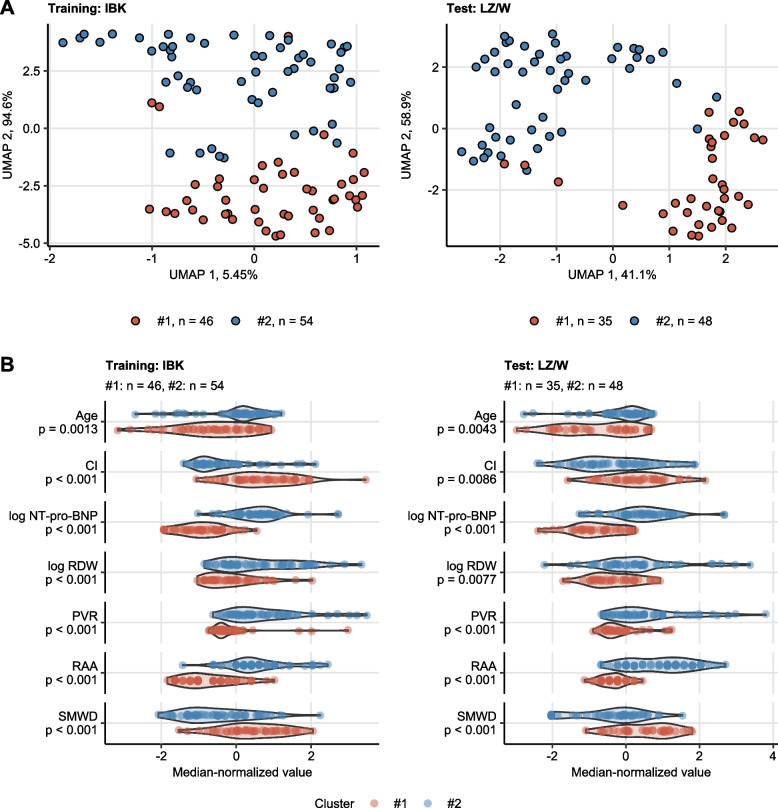
Clustering of the study participants. Clustering of the training Innsbruck (IBK) cohort participants in respect to the survival-associated factors identified by Elastic Net modeling (Fig. 2) was investigated by PAM (partition around medoids) algorithm with cosine distance. Numeric clustering features were median centered prior to the clustering. Cluster assignment in the training Linz/Vienna cohort (LZ/W) was done by an inverse distance weighted 7-nearest neighbor classifier. Numbers of individuals assigned to the PAH clusters are presented in the plot captions or legends. **A** PAH cluster assignment overlaid on the 2-dimensional cosine-distance UMAP (Uniform Manifold Approximation and Projection for Dimension Reduction) layout plots. Percentages of variance associated with the components are indicated in the plot axes. **B** Differences in the clustering features between the PAH clusters were assessed by Mann–Whitney test corrected for multiple testing with Benjamini–Hochberg method. Normalized, median-centered values of the clustering factors are shown in violin plots. Points represent single observations. *P* values are indicated in the Y axes. CI: cardiac index; NT-pro-BNP: N terminal pro brain natriuretic peptide; RDW: red blood cell distribution width; PVR: pulmonary vascular resistance; RAA: right atrial area; SMWD: six minute walking distance

Next, we investigated if the Elastic Net signature variables may be applied for definition of clinically relevant subsets of PAH. To this end, we assigned participants of the training IBK cohort to two clusters defined by the PAM algorithm with cosine distance [31, 32]⁠ in respect to age, CI, NT-pro-BNP, PVR, RAA, RDW and SMWD. The clustering algorithm of choice displayed the superior reproducibility in tenfold cross-validation [34]⁠ (accuracy = 0.97) and high explanatory value (‘explained’ variance fraction = 0.56) as compared with several other procedures such as hierarchical or k-means algorithm. The two cluster solution was also optimal in terms of explanatory value and reproducibility as tested for PAM/cosine distance clustering structures with varying cluster numbers. NT-pro-BNP, CI and RAA were found to be the most influential clustering factors (Supplementary Figures S4 – S5). The cluster assignment in the test LZ/W cohort was accomplished by a 7-nearest neighbors classifier (Fig. 4A). In both study collectives, the smaller PAH cluster #1 (IBK: 46%, LZ/W: 42% participants) encompassed significantly younger participants with better cardiac function reflected by a higher CI, lower RAA and lower blood NT-pro-BNP levels than cluster #2 participants. Furthermore, cluster #1 participants had lower PVR, lower RDW and superior SMWD in comparison with PAH cluster #2 (Fig. 4B). Additionally, mean pulmonary (mPAP) and right atrial (mRAP) pressure were significantly higher in PAH cluster #2 than in cluster #1 participants in both the IBK and LZ/W cohorts (Supplementary Figure S6, Supplementary Tables S4 – S5).

**Fig. 5:**
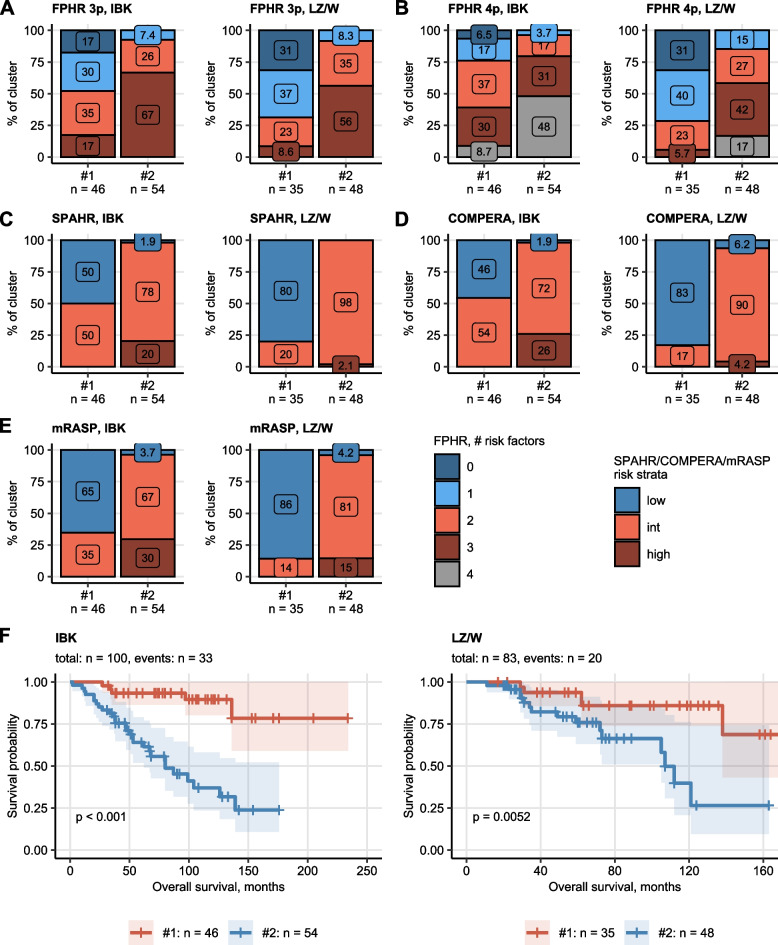
Risk assessment and survival differences in the PAH clusters. Risk assessment strata distribution and overall survival was compared between the study participant clusters with and log-rank test, respectively. *P* values were adjusted for multiple testing with Benjamini–Hochberg method. Numbers of individuals assigned to the clusters are presented in the Y axes or in the plot legends. **A – E** Risk assessment strata frequencies in the PAH clusters presented in stack plots. All differences were significant with *p* < 0.001. **F** Differences in overall survival in the PAH clusters visualized in Kaplan–Meier plots. *P* values are indicated in the plots. Numbers of complete observations and deaths are shown in the plot captions. FPHR 3p: French Pulmonary Hypertension Registry 3 parameter score, FPHR 4p: French Pulmonary Hypertension Registry 4 parameter score, COMPERA: Comparative, Prospective Registry of Newly Initiated Therapies for Pulmonary Hypertension score, mRASP: modified Risk Assessment Score of PAH; int.: intermediate

Finally, in line with the poorer physical performance and higher age, PAH cluster #2 demonstrated significantly worse risk profile composition with a higher number of risk factors in the FPHR model and more patients assigned to intermediate and high-risk strata by the mRASP, COMPERA and SPAHR tools. Consequently, overall survival in PAH cluster #2 was significantly shorter than in PAH cluster #1 (Fig. 5, Supplementary Figure S6, Supplementary Tables S5 – S6).

## Discussion

In PAH, standardized risk scores are used to evaluate the risk of mortality at initial presentation of the disease, and further to guide and adjust therapy upon a follow-up risk re-assessment and definition of treatment goals (e. g. low-risk status) [5]⁠. Consequently, accurate risk assessment is key to the successful PAH management and various risk assessment strategies have been proposed [18–20]⁠. While some approaches favor simple scores eligible for repetitive clinical evaluation, others employ numerous and sometimes not easily available parameter sets for high prediction accuracy [9]⁠. Interestingly, currently available risk scores are mainly based on expert opinion and traditional hypothesis-driven statistical tools such as logistic regression [9]⁠.

In this proof of concept study, we demonstrate the usefulness of supervised multi-parameter Elastic Net survival modeling [25, 26]⁠ and unsupervised PAM clustering [32]⁠ for search of novel biomarker combinations and subsets of PAH patients, which may improve PAH survival prediction. As PAH is a rare disease, patients from three Austrian PAH centers were included in our study. This design enabled us for an external validation of the modeling and clustering results. Our findings corroborate previous studies, reporting high accuracy of risk prediction and informative phenotyping of PAH patients by machine learning and clustering algorithms [15, 36, 37]⁠.

By Elastic Net regression, we generated a highly accurate and reproducible model, which outcompeted single demographic, biochemical and functional factors, such as the widely used COMPERA, SPAHR, mRASP and FPHR models [5, 8, 13, 21, 22]⁠ as well as the ensemble model combining those popular risk scales [26]⁠. The Elastic Net signature comprises well described risk factors of overall PAH mortality such as NT-pro-BNP, age, but also impaired functional parameters, including a reduced six-minute walking distance, as well as impaired hemodynamics, such as poor cardiac output and high pulmonary vascular resistance. However, those parameters are not consistently included in established risk scores [9, 15]⁠. Most prominently, advanced age, which was found to be a strong unmodifiable mortality predictor in the analyzed collectives both in the uni- and multi-variable setting, is not part of most risk assessment schemes including COMPERA, SPAHR, mRASP and FPHR. Another easily accessible parameter, RDW, was associated with poorer survival in Elastic Net modeling, which underscores the clinically relevant link between iron turnover, inflammation, oxidative stress, erythropoiesis and PAH progression [38–41]⁠. Of note, although the correlation of RDW and PAH mortality has been reported before [42]⁠, this risk factor is not routinely determined by automated blood count systems and has not been prospectively validated yet.

Our clustering scheme utilizing Elastic Net signature variables reproduces previously published phenotypes of typical and atypical PAH and demonstrates significant differences in the long-term follow-up of these patients [14]⁠. In detail, PAH cluster #2 encompassed mainly the elderly and, presumably, comorbid individuals with a shorter overall survival as compared to typical PAH patients preferentially assigned to cluster #1.

Our study has some limitations. First, as PAH is a rare condition, our study followed a multi-center retrospective design, which yielded only moderate size cohorts. Hence, the Elastic Net signature and PAH clustering scheme developed and tested in the IBK and LZ/W cohort need to be validated in larger prospective studies. A larger cohort size and algorithms robustly handling interactions between explanatory variables such as neuronal networks or tree models [36, 37]⁠ may improve the survival model accuracy even further. Second, although we screened a relatively large set of 19 explanatory factors compared with previous PAH risk analyses, we were not able to investigate some biomarkers of potential clinical relevance. For instance, diffusion capacity of carbon monoxide [15]⁠, was only available in the IBK cohort, and thus could not be included in the externally validated model. Additionally, we were not able to investigate new biomarkers, such as proteome profiling, as these parameters were not available for the presented cohorts [16, 17]⁠. Although implementation of such new parameters may improve survival prediction or identify subsets of PAH patients at higher risk, their usefulness is limited by costs, need for worldwide standardization and scarce medical resources. Thus, we herein focused on broadly available candidate risk factors, rather than entirely new biomarkers. Still, our Elastic Net signature may be easily expanded and adapted toadditional explanatory variables, thus providing a fast-track analysis tool for potential new biomarker sets in comparison to currently available risk models. Finally, our analysis included individuals at PAH diagnosis. Dynamics of the PAH clusters and modification of the risk predicted by Elastic Net modeling at follow-up and during treatment needs to be addressed by future research.

## Conclusions

**Fig. 6:**
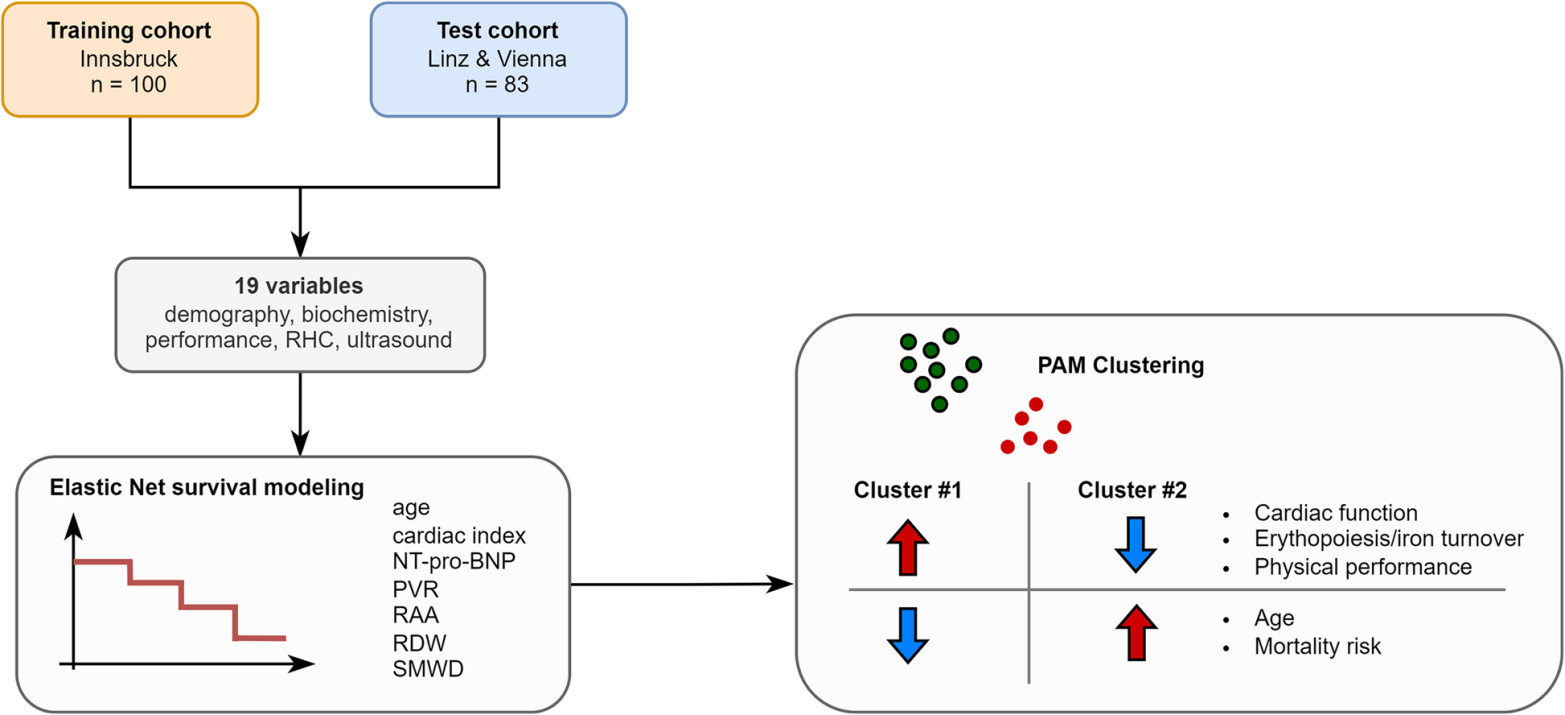
Summary of the analysis results. RHC: right heart catheterization; CI: cardiac index; NT-pro-BNP: N terminal pro brain natriuretic peptide; RDW: red blood cell distribution width; PVR: pulmonary vascular resistance; RAA: right atrial area; SMWD: six minute walking distance

We herein provide proof of principle, that supervised and unsupervised learning algorithms may improve risk assessment, and identify clinically relevant subsets of patients, hence contributing to a better understanding of biologically distinct PAH phenotypes (Fig. 6). This may pave the way to effective individualized risk management and treatment in PAH.

## Supplementary Information

Additional file 1.

## Data Availability

The study source data will be made available upon request. The source code of the analysis pipeline is available at https://github.com/PiotrTymoszuk/PAH-biomarker.

## Abbreviations

PAH: Pulmonary arterial hypertension
IBK: Innsbruck cohort
LZ/W: Linz/Vienna cohort
RHC: Right heart catheterization
mPAP: Mean pulmonary arterial pressure
PVR: Pulmonary vascular resistance
ESC/ESR: European Society of Cardiology/European Respiratory Society
GFR: Glomerular filtration rate
SO_2_: Peripheral hemoglobin oxygen saturation
FPHR: French Pulmonary Hypertension Registry
COMPERA: Prospective Registry of Newly Initiated Therapies for Pulmonary Hypertension
SPAHR: The Swedish PAH register
mRASP: Modified Risk Assessment Score of PAH
IQR: Interquartile range
C: C-index
IBS: Integrated Brier score
PAM: Partition around medoids
NT-pro-BNP: N-terminal pro-brain natriuretic peptide
SMWD: Six-minute walking distance
RAA: Right atrial area
CI: Cardiac index
RDW: Red blood cell distribution width
