# Supplementary material for: The combination of supervised and unsupervised learning based risk stratification and phenotyping in pulmonary arterial hypertension—a long-term retrospective multicenter trial
Source: BMC Pulm Med. 2023 Apr 25;23:143. doi: 10.1186/s12890-023-02427-2 (PMC10131314; doi:10.1186/s12890-023-02427-2)
Supplement: Supplementary file 1 — Additional file 1. [file 12890_2023_2427_MOESM1_ESM.pdf]

The Combination of Supervised and Unsupervised Learning based Risk Stratification and Phenotyping in Pulmonary Arterial Hypertension - a Long-term Retrospective Multicenter Trial

Supplementary Material

Innsbruck PAH registry

2023-03-27

Supplementary Methods

Software

Data transformation, analysis and result visualization was accomplished by R version 4.2.3.

Import of the study data was accomplished with the *readxl* package [1]. Tabular data were handled by the *tidyverse* package bundle [2] and the packages *rlang* [3] and *trafo*. Text data were handled with the *stringi* package [4].

Exploratory data analysis and statistical hypothesis for numeric and categorical variables testing was done with *rstatix* [5], *DescTools* [6] and *ExDA*. Survival analysis was accomplished with the packages *survival* [7], *survminer* [8], *pec* [9], *rms* [10], *glmnet* [11], *survmisc* [12] and the development package *coxExtensions*. Cross-validation folds were generated with *caret* [13]. Clustering analysis was done with the packages *cluster* [14], *philentropy* [15], *factoextra* [16], *umap* [17] and *clustTools*.

Results were visualized with *ggplot2* [2] (plots of model estimates, violin, bubble, bar and Forest plots), *ExDA* (stack plots, plots of effect sizes/significance of differences between clusters), *survminer* and *coxExtensions* (Kaplan-Meier plots) and *clustTools* (UMAP layout plots, cluster diagnostic plots). Tables were generated with *flextable* [18], figures were created with *cowplot* [19].

The Supplementary Material file was built with *rmarkdown* environment [20] with the packages *knitr* [21] and *bookdown* [22].

Data import and transformation, visualization, descriptive statistic

The study dataset was imported into from an Excel file (function `read_excel()`, package *readxl*) and formatted with an in-house developed script.

For univariable survival modeling and construction of candidate risk signatures, a set of categorical 19 demographic, biochemical, right-heart catheter, laboratory, ultrasound and lung function parameters recorded at PH diagnosis was used. To improve normality of some independent variables (NT-pro-BNP, RDW, TF-Sat, ferritin) prior to survival modeling, log transformation was applied.

Prior to modeling and clustering, numeric variables were normalized and median-centered (function `scale(x, center = median(x))`). To account for non-linear associations of numeric independent variables with survival, both 1st and 2nd order terms were included in

the Cox models. In modeling, categorical explanatory variables were converted to dummy numeric variables (function `model.matrix()`).

For the list of modeling variables and their transformation and stratification scheme, see: **Supplementary Table S1**.

Descriptive statistic, hypothesis testing, multiple comparisons

Numeric variables are presented as medians with interquartile ranges (IQR). Categorical variables are shown as percentages and counts within the complete observation set for each variable category (function `explore()`, package *ExDA*).

As the some of the analyzed numeric variables were non-normally distributed as checked by Shapiro-Wilk test (function `explore(what = 'normality')`, package *ExDA*), differences in median values of numeric variables between the study cohorts or participant clusters were investigated by Mann-Whitney test and r effect size statistic. Differences in frequency distribution of categorical variables between the study cohorts or participant clusters were assessed by χ^2 test and Cramer's V effect size statistic. Statistical significance for the comparisons was determined with the function `compare_variables()` from the *ExDA* package.

Risk strata returned by established PAH risk assessment tools (mRASP: modified risk assessment score of PAH; COMPERA: comparative, prospective registry of newly initiated therapies for pulmonary hypertension score; SPAHR: Swedish pulmonary arterial hypertension registry score; FPHR 3p: french pulmonary arterial hypertension registry score; FPHR 4p: french pulmonary arterial hypertension registry score, 4 parameters) was converted to numeric values (mRASP/COMPERA/SPAHR: low: 1, intermediate: 2, high risk: 3, FPHR scales: number of risk factors). Their correlation with the newly developed Elastic Net signature was assessed by Spearman's test (function `correlate_variables(type = 'spearman')`, package *ExDA*). Consistency of the entire risk assessment battery available for both study cohorts (Elastic Net, mRASP, COMPERA, SPAHR, FPHR 3p, FPHR 4p) was assessed by Kendall's coefficient of concordance (KCC; computed with `KendallW()`, package *DescTools*).

Differences in survival between the participant clusters or participants stratified by risk score tertiles or by the PAH cluster assignment were compared by Kaplan-Meier (KM) analysis with log-rank test [7, 8, 23] (functions `surv_fit()` and `surv_pvalue()`, package *survminer*). Fractions of surviving participants were presented in Kaplan-Meier plots (function `ggsurvplot()`, package *survminer*).

For each analysis task and cohort, p values were corrected for multiple comparisons with Benjamini-Hochberg method [24].

Univariable Cox survival modeling

Association of independent categorical and numeric variables (**Supplementary Table S1**) with overall survival time was assessed by series of univariable Cox proportional hazard models constructed for the Innsbruck and Linz/Vienna cohort using the *survival* [7] and *coxExtensions* packages (functions `coxph()` and `as_coxex()`). Significance of the hazard ratio estimates was determined by Wald Z test. P values were corrected for multiple comparisons with Benjamini-Hochberg method [24]. Proportional hazard assumption was checked with the `summary(type = 'assumptions')` method (package *coxExtensions*, a wrapper around `cox.zph()` from `survival`) [25]. Concordance indexes (C-index) [26], integrated Brier scores (IBS) [27] and R^2 [12] served as measures of predictive performance of the Cox models and were computed with the `summary(type = 'fit')` method (package *coxExtensions*, employing the `concordance()`, `pec()` and `rsq()` functions from the packages *survival*, *pec* and *survMisc*).

For the full modeling results, see: **Supplementary Table S3**.

Multivariable Cox survival modeling with the Elastic Net technique

Multi-parameter Cox modeling with the set of independent categorical and numeric variables (**Supplementary Table S1**) was accomplished by Elastic Net machine learning technique [11, 28]. The Elastic Net Cox proportional hazard model was trained in the Innsbruck cohort (function `glmnet(alpha = 0.5, family = 'cox')`, package *glmnet*). The optimal lambda parameter (λ = 0.166) for the training cohort model construction was found by 200-repetition 10-fold cross-validation (function `cv.glmnet()`, package *glmnet*) and corresponded to the minimum of cross-validation error (`lambda.min` parameter). Non-zero Elastic Net model coefficients termed further 'Elastic Net signature' are presented in **Figure 2A**. Subsequently, the Elastic Net model linear predictor (LP) scores were calculated for the training IBK and test Linz/Vienna cohort and their association with overall survival was assessed by univariable Cox modeling [29]. C-index, IBS and R^2 served as performance measures as described above for univariable Cox modeling [12, 26, 27] (**Supplementary Table S4**). Differences in survival between study participants stratified by the LP score tertiles were assessed by log-rank test as described above [8, 23].

Ridge ensemble model

Multi-parameter Cox model including the established PAH risk assessment tools (mRASP, SPAHR, COMPERA: low, intermediate and high risk strata, FPHR 3p and FPHR 4p: number of risk factors coded as categorical variable) as explanatory variables was constructed with the Ridge Cox regression algorithm [11, 28]. The Ridge ensemble model was trained in the Innsbruck cohort (function `glmnet(alpha = 0, family = 'cox')`, package *glmnet*). The

optimal lambda parameter (λ = 0.0617) for the training cohort model construction was found by 200-repetition 10-fold cross-validation (function `cv.glmnet()`, package *glmnet*) and corresponded to the minimum of cross-validation error (`lambda.min` parameter). The values of non-zero Ridge model coefficients are presented in **Supplementary Figure S3A**. Subsequently, the Ridge model linear predictor (LP) scores were calculated for the training IBK and test Linz/Vienna cohort and their association with overall survival was assessed by univariable Cox modeling [29] analogically to the Elastic Net signature described above. Performance metrics of the Ridge ensemble are presented in **Supplementary Table S4**.

Comparison of risk assessment tools

Univariable Cox models were constructed for the Elastic Net signature and the Ridge ensemble described above and for the established PAH risk assessment tools available for both cohorts (mRASP, SPAHR, COMPERA: low, intermediate and high risk strata, FPHR 3p and FPHR 4p: number of risk factors coded as categorical variable) as described for univariable Cox modeling. Performance metrics: C-indexes, IBS and R^2 are presented in **Supplementary Table S4**.

Clustering of the study participants

Clustering of the study participants in the Innsbruck training cohort in respect to the variables found associated with overall survival by the Elastic Net Cox modeling (**Figure 2A**; Age, SMWD, log RDW, CI, PVR, log NT-pro-BNP, RAA) was done with the PAM algorithm (partitioning around medoids) [14] with the cosine distance between the study participants [15] (clustering objects generated with `kcluster(distance_method = 'cosine', clust_fun = 'pam', k = 2)`, package *clustTools*). The PAM/cosine distance clustering procedure demonstrated the superior fraction of 'explained' clustering variance (ratio of total between cluster sum of squared to total sum of squares) and the optimal performance in 10-fold cross-validation measured by the fraction of correct cluster assignments [30] as compared with hierarchical clustering, k-means and self-organizing map algorithms (**Supplementary Figure S4A**). Clustering variances and CV accuracy were computed with the `var()` and `cv()` methods provided by the *clustTools* called for the clustering objects.

The choice of cluster number k = 2 was based on the bend of the within-cluster sum-of-squares curve, the peak value of silhouette statistic [16, 31] and optimal cluster assignment accuracy in 10-fold CV assessed for the PAM/cosine distance clustering structures generated for multiple k values (methods `plot()` and `cv()`, package *clustTools*) (**Supplementary Figure S4B**).

The importance of specific clustering features was determined by comparing the 'explained' clustering variances of the original clustering structure with the clustering

objects with randomly re-shuffled clustering features [32] with the `impact()` function from the *clustTools* package.

Assignment of the test Linz/Vienna cohort participants to the PAH clusters defined in the Innsbruck cohort was done with an inverse distance-weighted 7-nearest neighbor label propagation procedure [32–34]. This semi-supervised clustering procedure yielded clusters of comparable sized in both cohorts (Innsbruck: #1: n = 46, #2: n = 54; Linz/Vienna: #1: n = 35, #2: n = 48). The ratios of 'explained' clustering variances were 0.56 and 0.36 for the training Innsbruck cohort and the test Linz/Vienna collective, respectively. Assignment of participants to the PAH clusters was visualized with two-dimensional cosine distance UMAP (Uniform Manifold Approximation and Projection, function `plot(type = 'components', red_fun = 'umap', kdim = '2', with = 'data')`, package *clustTools*) [17, 35].

Differences in study variables between the participant clusters were determined by Mann-Whitney or χ^2 test as described above (**Supplementary Tables S5 - S5**). Differences in overall survival between the clusters were compared with KM method and log-rank test as described above.

Data and code availability

The study data set is available at serious request to the corresponding author. The analysis R code was deposited on GitHub (https://github.com/PiotrTymoszuk/PAH-biomarker).

Supplementary Tables

Supplementary Table S1: Study variables.

Name in R	Description	Name in the report	Unit	Stratification	Used in risk modeling
age_fc	age at the diagnosis	Age	years		yes
SMWD	Six Minute Walk Distance	SMWD	m		yes
mPAP	Mean pulmonary arterial pressure	mPAP	mmHg		yes
event3	3-year mortality	3-year mortality		no; yes	no
event5	5-year mortality	5-year mortality		no; yes	no
death_study	overall mortality during the study period	Overall mortality			no
death_study_fct	overall mortality during the study period	Overall mortality		no; yes	no
surv_months	survival time from the diagnosis	OS	months		no
sex	sex	Sex		female; male	yes
PVR	Pulmonary vascular resistance	PVR	Wood		yes
PCWP	Pulmonary capillary wedge pressure	PCWP	mmHg		yes
anemia	anemia	Anemia		no; yes	yes
RDW_log	red blood cell distribution width	log RDW	%		yes
renal_ins	Renal insufficiency, GFR < 60%	Renal insufficiency		no; yes	yes
FT_log	Ferritin	log FT	ng/ml		yes
TSAT_log	Transferrin saturation	log TF-Sat	%		yes
MCV	Mean corpuscular volume	MCV	fl		yes

**Name in R**	**Description**	**Name in the report**	**Unit**	**Stratification**	**Used in risk modeling**
NTproBNP_log	N-terminal pro-brain natriuretic peptide	log NT-pro-BNP	pg/ml		yes
percardial_effusion	Percardial effusion	Percardial effusion		no; yes	yes
RA_area	Right Atrial Area	RAA	cm2		yes
cardiac_index	Cardiac index	CI			yes
mRAP	Mean right atrial pressure	mRAP	mmHg		yes
WHOFc_class	WHO Functional Classification	WHO class		I/II; III/IV	yes
SO2_RL_class	O2 saturation	SO2	%	≥95; <95	yes
mRASP	modified Risk Assessment Score of PAH	mRASP		low; int; high	no
Compera	Comparative, Prospective Registry of Newly Initiated Therapies for Pulmonary Hypertension score	COMPERA		low; int; high	no
SPAHR	Swedish Pulmonary Arterial Hypertension Registry score	SPAHR		low; int; high	no
FRENCH3p	French Pulmonary Arterial Hypertension Registry score, 3 parameters	FPHR 3p		0; 1; 2; 3	no
FRENCH4p	French Pulmonary Arterial Hypertension Registry score, 4 parameters	FPHR 4p		0; 1; 2; 3; 4	no
Reveal_lite2_3_cat	Reveal Lite, Risk Classes	Reveal Lite		low; int; high	no
Reveal2_risk_3_cat	Reveal 2.0 Risk classes	Reveal 2.0		low; int; high	no

Supplementary Table S2: Supplementary characteristic of the study cohorts. Numeric variables are presented as medians with interquartile ranges (IQR) and ranges. Categorical variables are presented as percentages and counts within the complete observation set.

Variable[a]	IBK	LZ/W	Significance[b]	Effect size[b]
Participants, n	100	83		
mRAP, mmHg	10 [IQR: 6 - 13]<br>2 - 26	6 [IQR: 3 - 9]<br>0 - 20	p < 0.001	r = 0.43
SO2, %	≥95: 47% (47)<br><95: 53% (53)	≥95: 48% (40)<br><95: 52% (43)	ns (p = 0.99)	V = 0.012
log NT-pro-BNP, pg/ml	6.6 [IQR: 5.1 - 7.7]<br>3.4 - 11	6.6 [IQR: 5.3 - 7.4]<br>3.2 - 10	ns (p = 0.95)	r = 0.0079
CI	2.4 [IQR: 1.9 - 2.8]<br>1.6 - 4.2	2.6 [IQR: 2.2 - 3]<br>1.4 - 3.8	p = 0.033	r = 0.19
RAA, cm^2	22 [IQR: 17 - 24]<br>13 - 34	18 [IQR: 17 - 23]<br>13 - 30	p = 0.014	r = 0.22
MCV, fl	88 [IQR: 85 - 91]<br>58 - 100	89 [IQR: 86 - 93]<br>76 - 110	ns (p = 0.33)	r = 0.1
log RDW, %	2.7 [IQR: 2.6 - 2.8]<br>2.5 - 3.1	2.7 [IQR: 2.7 - 2.8]<br>2.5 - 3.1	p = 0.014	r = 0.22
log FT, ng/ml	4.1 [IQR: 3.5 - 4.7]<br>1.1 - 6.5	4.4 [IQR: 3.4 - 4.9]<br>1.9 - 7.1	ns (p = 0.6)	r = 0.056
log TF-Sat, %	3 [IQR: 2.6 - 3.3]<br>0.69 - 4.3	3 [IQR: 2.5 - 3.4]<br>0.69 - 4.5	ns (p = 0.52)	r = 0.066
mRASP, risk strata	low: 32% (32)<br>int: 52% (52)<br>high: 16% (16)	low: 39% (32)<br>int: 53% (44)<br>high: 8.4% (7)	ns (p = 0.4)	V = 0.12
COMPERA, risk strata	low: 22% (22)<br>int: 64% (64)<br>high: 14% (14)	low: 39% (32)<br>int: 59% (49)<br>high: 2.4% (2)	p = 0.014	V = 0.25
SPAHR, risk strata	low: 24% (24)<br>int: 65% (65)<br>high: 11% (11)	low: 34% (28)<br>int: 65% (54)<br>high: 1.2% (1)	p = 0.043	V = 0.21
FPHR 3p, number of risk factors	0: 8% (8)<br>1: 18% (18)<br>2: 30% (30)	0: 13% (11)<br>1: 20% (17)<br>2: 30% (25)	ns (p = 0.72)	V = 0.11

Variable[a]	IBK	LZ/W	Significance[b]	Effect size[b]
	3: 44% (44)	3: 36% (30)		
FPHR 4p, number of risk factors	0: 3% (3)<br>1: 10% (10)<br>2: 26% (26)<br>3: 31% (31)<br>4: 30% (30)	0: 13% (11)<br>1: 25% (21)<br>2: 25% (21)<br>3: 27% (22)<br>4: 9.6% (8)	p = 0.002	V = 0.35
3-year mortality	13% (13)	11% (9)	ns (p = 0.94)	V = 0.033
Overall mortality	33% (33)	24% (20)	ns (p = 0.38)	V = 0.098

[a]mRAP: mean right atrial pressure; SO2: oxygen saturation; NT-pro-BNP: N-terminal pro-brain natriuretic peptide; CI: cardiac index; RAA: right atrial area; MCV: mean corpuscular volume; RDW: red blood cell distribution width; FT: ferritin; mRASP: modified risk assessment score of PAH; COMPERA: comparative, prospective registry of newly initiated therapies for pulmonary hypertension score; SPAHR: Swedish pulmonary arterial hypertension registry score; FPHR 3p: french pulmonary arterial hypertension registry score, 3 parameters; FPHR 4p: french pulmonary arterial hypertension registry score, 4 parameters.

[b]Numeric variables: Mann-Whitney U test with r effect size statistic; categorical variables: χ^2 test with Cramer V effect size statistic; survival: log-rank test.

Supplementary Table S3: Results of univariable Cox modeling in the Innsbruck (IBK) and Linz/Vienna (LZ/W) cohort

Cohort	Variable[a]	Level	Order	HR, 95% CI[b]	Significance	C index, 95% CI[c]	R^2	IBS[d]
IBK	Age		1	2.6 [1.3 - 4.9]	p = 0.016	0.68 [0.59 - 0.78]	0.33	0.11
			2	0.72 [0.36 - 1.5]	ns (p = 0.45)	0.68 [0.59 - 0.78]	0.33	0.11
	SMWD		1	0.41 [0.24 - 0.7]	p = 0.0068	0.7 [0.59 - 0.8]	0.3	0.17
			2	0.82 [0.56 - 1.2]	ns (p = 0.4)	0.7 [0.59 - 0.8]	0.3	0.17
	mPAP		1	4.6 [2.2 - 9.4]	p < 0.001	0.74 [0.65 - 0.83]	0.38	0.14
			2	0.63 [0.45 - 0.88]	p = 0.02	0.74 [0.65 - 0.83]	0.38	0.14
	Sex	male		2 [1 - 4]	ns (p = 0.12)	0.61 [0.52 - 0.7]	0.069	0.18
	PVR		1	3.5 [1.6 - 7.8]	p = 0.0095	0.68 [0.59 - 0.78]	0.25	0.18
			2	0.73 [0.52 - 1]	ns (p = 0.15)	0.68 [0.59 - 0.78]	0.25	0.18
	PCWP		1	1.3 [0.76 - 2.2]	ns (p = 0.45)	0.51 [0.41 - 0.61]	0.017	0.18
			2	0.92 [0.71 - 1.2]	ns (p = 0.59)	0.51 [0.41 - 0.61]	0.017	0.18
	Anemia	yes		1.4 [0.59 - 3.2]	ns (p = 0.55)	0.54 [0.46 - 0.62]	0.0093	0.2
	log RDW		1	1.8 [0.96 - 3.3]	ns (p = 0.14)	0.66 [0.55 - 0.77]	0.2	0.18
			2	0.99 [0.75 - 1.3]	ns (p = 0.92)	0.66 [0.55 - 0.77]	0.2	0.18
	Renal insufficiency	yes		2.5 [1.3 - 5]	p = 0.021	0.6 [0.51 - 0.69]	0.13	0.17
	log FT		1	1.3 [0.88 - 1.9]	ns (p = 0.3)	0.6 [0.49 - 0.71]	0.039	0.18
			2	0.93 [0.72 - 1.2]	ns (p = 0.59)	0.6 [0.49 - 0.71]	0.039	0.18
	log TF-Sat		1	1 [0.7 - 1.5]	ns (p = 0.9)	0.58 [0.48 - 0.68]	0.056	0.2
			2	1.2 [0.97 - 1.4]	ns (p = 0.16)	0.58 [0.48 - 0.68]	0.056	0.2
	MCV		1	1.4 [1 - 2.1]	ns (p = 0.12)	0.54 [0.41 - 0.66]	0.13	0.18
			2	1.2 [1.1 - 1.3]	p = 0.0087	0.54 [0.41 - 0.66]	0.13	0.18
	log NT-pro-BNP		1	3.9 [2 - 7.7]	p < 0.001	0.76 [0.67 - 0.85]	0.49	0.11
			2	0.58 [0.4 - 0.84]	p = 0.013	0.76 [0.67 - 0.85]	0.49	0.11

Cohort	Variable[a]	Level	Order	HR, 95% CI[b]	Significance	C index, 95% CI[c]	R^2	IBS[d]
	Percardial effusion	yes		2.1 [0.91 - 4.9]	ns (p = 0.15)	0.56 [0.48 - 0.63]	0.048	0.19
	RAA		1	3.6 [1.8 - 7.2]	p = 0.0034	0.73 [0.65 - 0.81]	0.43	0.12
			2	0.46 [0.29 - 0.74]	p = 0.0068	0.73 [0.65 - 0.81]	0.43	0.12
	CI		1	0.31 [0.19 - 0.52]	p < 0.001	0.77 [0.68 - 0.85]	0.4	0.15
			2	1.3 [0.84 - 2]	ns (p = 0.35)	0.77 [0.68 - 0.85]	0.4	0.15
	mRAP		1	1.5 [0.93 - 2.5]	ns (p = 0.16)	0.56 [0.46 - 0.66]	0.074	0.17
			2	0.92 [0.72 - 1.2]	ns (p = 0.59)	0.56 [0.46 - 0.66]	0.074	0.17
	WHO class	III/IV		1.9 [0.86 - 4]	ns (p = 0.18)	0.55 [0.46 - 0.64]	0.05	0.19
	SO2	<95		1.5 [0.76 - 3.1]	ns (p = 0.34)	0.58 [0.49 - 0.67]	0.027	0.19
LZ/W	Age		1	3.8 [1.2 - 12]	ns (p = 0.076)	0.69 [0.56 - 0.82]	0.26	0.13
			2	1.4 [0.78 - 2.5]	ns (p = 0.43)	0.69 [0.56 - 0.82]	0.26	0.13
	SMWD		1	0.44 [0.24 - 0.8]	p = 0.048	0.68 [0.56 - 0.8]	0.3	0.14
			2	0.93 [0.62 - 1.4]	ns (p = 0.79)	0.68 [0.56 - 0.8]	0.3	0.14
	mPAP		1	1.9 [1.1 - 3.4]	ns (p = 0.086)	0.63 [0.5 - 0.76]	0.19	0.15
			2	0.81 [0.53 - 1.2]	ns (p = 0.49)	0.63 [0.5 - 0.76]	0.19	0.15
	Sex	male		5.7 [2.2 - 15]	p = 0.011	0.73 [0.64 - 0.83]	0.33	0.12
	PVR		1	5.8 [2 - 16]	p = 0.011	0.74 [0.61 - 0.87]	0.38	0.15
			2	0.54 [0.33 - 0.87]	ns (p = 0.064)	0.74 [0.61 - 0.87]	0.38	0.15
	PCWP		1	0.73 [0.31 - 1.7]	ns (p = 0.55)	0.61 [0.46 - 0.76]	0.17	0.15
			2	0.48 [0.2 - 1.1]	ns (p = 0.25)	0.61 [0.46 - 0.76]	0.17	0.15
	Anemia	yes		1.9 [0.69 - 5.4]	ns (p = 0.4)	0.52 [0.43 - 0.6]	0.042	0.15
	log RDW		1	1.7 [0.91 - 3]	ns (p = 0.25)	0.61 [0.47 - 0.74]	0.1	0.15
			2	0.93 [0.69 - 1.2]	ns (p = 0.68)	0.61 [0.47 - 0.74]	0.1	0.15
	Renal insufficiency	yes		1.5 [0.49 - 4.5]	ns (p = 0.55)	0.49 [0.41 - 0.57]	0.014	0.16

Cohort	Variable[a]	Level	Order	HR, 95% CI[b]	Significance	C index, 95% CI[c]	R^2	IBS[d]
	log FT		1	1.4 [0.93 - 2.1]	ns (p = 0.26)	0.58 [0.43 - 0.72]	0.075	0.15
			2	1.1 [0.88 - 1.4]	ns (p = 0.54)	0.58 [0.43 - 0.72]	0.075	0.15
	log TF-Sat		1	0.61 [0.34 - 1.1]	ns (p = 0.25)	0.61 [0.47 - 0.75]	0.097	0.14
			2	0.86 [0.59 - 1.3]	ns (p = 0.55)	0.61 [0.47 - 0.75]	0.097	0.14
	MCV		1	1.4 [0.82 - 2.5]	ns (p = 0.4)	0.62 [0.46 - 0.78]	0.059	0.15
			2	0.84 [0.54 - 1.3]	ns (p = 0.55)	0.62 [0.46 - 0.78]	0.059	0.15
	log NT-pro-BNP		1	2.2 [1.3 - 3.6]	p = 0.02	0.67 [0.52 - 0.82]	0.31	0.12
			2	0.96 [0.69 - 1.3]	ns (p = 0.83)	0.67 [0.52 - 0.82]	0.31	0.12
	Percardial effusion	yes		4 [0.89 - 18]	ns (p = 0.22)	0.53 [0.47 - 0.59]	0.071	0.15
	RAA		1	1.5 [0.66 - 3.5]	ns (p = 0.49)	0.65 [0.53 - 0.78]	0.12	0.15
			2	1 [0.62 - 1.7]	ns (p = 0.95)	0.65 [0.53 - 0.78]	0.12	0.15
	CI		1	0.63 [0.35 - 1.1]	ns (p = 0.27)	0.68 [0.55 - 0.82]	0.087	0.16
			2	0.84 [0.56 - 1.3]	ns (p = 0.54)	0.68 [0.55 - 0.82]	0.087	0.16
	mRAP		1	2.4 [1.2 - 4.8]	ns (p = 0.072)	0.72 [0.59 - 0.84]	0.25	0.15
			2	0.79 [0.52 - 1.2]	ns (p = 0.43)	0.72 [0.59 - 0.84]	0.25	0.15
	WHO class	III/IV		5.5 [2 - 15]	p = 0.011	0.69 [0.58 - 0.79]	0.36	0.13
	SO2	<95		1.7 [0.71 - 4.3]	ns (p = 0.4)	0.56 [0.43 - 0.68]	0.045	0.15

[a]SMWD: six-minute walking distance; mPAP: mean pulmonary arterial pressure; PVR: pulmonary vascular resistance; PCWP: pulmonary capillary wedge pressure; RDW: red blood cell distribution width; FT: ferritin; TF-Sat: transferrin saturation; MCV: mean corpuscular volume; NT-pro-BNP: N-terminal pro-brain natriuretic peptide; RAA: right atrial area; CI: cardiac index; WHO class: WHO functional class; SO2: oxygen saturation.

[b]HR, 95% CI: hazard ratio with 95% confidence intervals.

[c]C index, 95% CI: concordance index with 95% confidence intervals.

[d]IBS: integrated Brier score.

Supplementary Table S4: Performance of the Elastic Net signature and established risk assessment tools at predicting overall PAH survival.

**Cohort**	**Variable**[a]	**C index, 95% CI**[b]	**R^2**	**IBS**[c]
IBK	ElasticNet	0.82 [0.75 - 0.89]	0.66	0.098
	Ridge ensemble	0.79 [0.71 - 0.86]	0.58	0.095
	mRASP	0.72 [0.64 - 0.8]	0.44	0.12
	COMPERA	0.7 [0.63 - 0.78]	0.41	0.13
	SPAHR	0.73 [0.66 - 0.79]	0.51	0.11
	FPHR 3p	0.66 [0.58 - 0.74]	0.37	0.14
	FPHR 4p	0.69 [0.61 - 0.77]	0.36	0.13
LZ/W	ElasticNet	0.77 [0.66 - 0.88]	0.52	0.11
	Ridge ensemble	0.72 [0.6 - 0.84]	0.39	0.12
	mRASP	0.67 [0.59 - 0.76]	0.37	0.11
	COMPERA	0.64 [0.54 - 0.74]	0.23	0.14
	SPAHR	0.66 [0.57 - 0.75]	0.29	0.13
	FPHR 3p	0.69 [0.58 - 0.8]	0.38	0.13
	FPHR 4p	0.75 [0.65 - 0.86]	0.34	0.14

[a]Ridge ensemble: Ridge Cox regression model with the established risk assessment scales; mRASP: modified risk assessment score of PAH; COMPERA: comparative, prospective registry of newly initiated therapies for pulmonary hypertension score; SPAHR: Swedish pulmonary arterial hypertension registry score; FPHR 3p: french pulmonary arterial hypertension registry score, 3 parameters; FPHR 4p: french pulmonary arterial hypertension registry score, 4 parameters.

[b]C index, 95% CI: concordance index with 95% confidence intervals.

[c]IBS: integrated Brier score.

Supplementary Table S5: Characteristic of the participant clusters in the Innsbruck cohort. Numeric variables are presented as medians with interquartile ranges (IQR) and ranges. Categorical variables are presented as percentages and counts within the complete observation set.

**Variable**[a]	**Cluster #1**	**Cluster #2**	**Significance**[b]	**Effect size**[b]
Participants, n	46	54		
3-year mortality	6.5% (3)	19% (10)	ns (p = 0.16)	V = 0.18
5-year mortality	6.5% (3)	33% (18)	p = 0.0039	V = 0.33
Overall mortality	11% (5)	52% (28)	p < 0.001	V = 0.43
Age, years	58 [IQR: 48 - 68]<br>19 - 80	69 [IQR: 65 - 74]<br>26 - 84	p = 0.0013	r = 0.34
SMWD, m	370 [IQR: 310 - 450]<br>120 - 580	240 [IQR: 160 - 330]<br>50 - 610	p < 0.001	r = 0.47
mPAP, mmHg	30 [IQR: 27 - 37]<br>26 - 87	47 [IQR: 40 - 55]<br>26 - 120	p < 0.001	r = 0.56
Sex	female: 78% (36)<br>male: 22% (10)	female: 52% (28)<br>male: 48% (26)	p = 0.017	V = 0.27
PVR, Wood	6.8 [IQR: 5.9 - 9.6]<br>3.3 - 38	14 [IQR: 11 - 22]<br>4.3 - 43	p < 0.001	r = 0.6
PCWP, mmHg	12 [IQR: 9 - 14]<br>6 - 23	14 [IQR: 11 - 19]<br>4 - 32	p = 0.041	r = 0.22
Anemia	15% (7)	22% (12)	ns (p = 0.53)	V = 0.089
log RDW, %	2.6 [IQR: 2.6 - 2.7]<br>2.5 - 2.9	2.7 [IQR: 2.6 - 2.8]<br>2.6 - 3.1	p < 0.001	r = 0.38
Renal insufficiency	17% (8)	50% (27)	p = 0.0024	V = 0.34
log FT, ng/ml	4 [IQR: 3.3 - 4.7]<br>1.1 - 6.5	4.2 [IQR: 3.8 - 4.9]<br>1.8 - 6.5	ns (p = 0.23)	r = 0.12
log TF-Sat, %	3 [IQR: 2.7 - 3.4]<br>1.8 - 4.3	2.8 [IQR: 2.4 - 3.2]<br>0.69 - 4	ns (p = 0.16)	r = 0.15
MCV, fl	88 [IQR: 85 - 90]<br>76 - 96	88 [IQR: 86 - 92]<br>58 - 100	ns (p = 0.33)	r = 0.1
log NT-pro-BNP, pg/ml	5.1 [IQR: 4.4 - 5.8]	7.6 [IQR: 6.9 - 8.1]	p < 0.001	r = 0.78

Variable[a]	Cluster #1	Cluster #2	Significance[b]	Effect size[b]
	3.4 - 7.5	4.9 - 11		
Percardial effusion	6.5% (3)	24% (13)	p = 0.046	V = 0.24
RAA, cm^2	17 [IQR: 16 - 21]<br>13 - 27	24 [IQR: 23 - 27]<br>15 - 34	p < 0.001	r = 0.67
CI, NA	2.7 [IQR: 2.4 - 3]<br>1.8 - 4.2	2 [IQR: 1.9 - 2.3]<br>1.6 - 3.5	p < 0.001	r = 0.6
mRAP, mmHg	8 [IQR: 6 - 12]<br>2 - 18	11 [IQR: 8 - 14]<br>2 - 26	p = 0.023	r = 0.24
WHO class	I/II: 48% (22)<br>III/IV: 52% (24)	I/II: 31% (17)<br>III/IV: 69% (37)	ns (p = 0.16)	V = 0.17
SO2	≥95: 57% (26)<br><95: 43% (20)	≥95: 39% (21)<br><95: 61% (33)	ns (p = 0.15)	V = 0.18
mRASP, risk strata	low: 65% (30)<br>int: 35% (16)<br>high: 0% (0)	low: 3.7% (2)<br>int: 67% (36)<br>high: 30% (16)	p < 0.001	V = 0.69
COMPERA, risk strata	low: 46% (21)<br>int: 54% (25)<br>high: 0% (0)	low: 1.9% (1)<br>int: 72% (39)<br>high: 26% (14)	p < 0.001	V = 0.59
SPAHR, risk strata	low: 50% (23)<br>int: 50% (23)<br>high: 0% (0)	low: 1.9% (1)<br>int: 78% (42)<br>high: 20% (11)	p < 0.001	V = 0.6
FPHR 3p, number of risk factors	0: 17% (8)<br>1: 30% (14)<br>2: 35% (16)<br>3: 17% (8)	0: 0% (0)<br>1: 7.4% (4)<br>2: 26% (14)<br>3: 67% (36)	p < 0.001	V = 0.56
FPHR 4p, number of risk factors	0: 6.5% (3)<br>1: 17% (8)<br>2: 37% (17)<br>3: 30% (14)<br>4: 8.7% (4)	0: 0% (0)<br>1: 3.7% (2)<br>2: 17% (9)<br>3: 31% (17)<br>4: 48% (26)	p < 0.001	V = 0.5
Reveal Lite, risk strata	low: 70% (32)<br>int: 17% (8)<br>high: 13% (6)	low: 5.6% (3)<br>int: 11% (6)<br>high: 83% (45)	p < 0.001	V = 0.73
Reveal 2.0, risk strata	low: 65% (30)<br>int: 17% (8)	low: 3.7% (2)<br>int: 11% (6)	p < 0.001	V = 0.72

Variable[a]	Cluster #1	Cluster #2	Significance[b]	Effect size[b]
	high: 17% (8)	high: 85% (46)		

[a]SMWD: six-minute walking distance; mPAP: mean pulmonary arterial pressure; PVR: pulmonary vascular resistance; PCWP: pulmonary capillary wedge pressure; RDW: red blood cell distribution width; FT: ferritin; TF-Sat: transferrin saturation; MCV: mean corpuscular volume; NT-pro-BNP: N-terminal pro-brain natriuretic peptide; RAA: right atrial area; CI: cardiac index; WHO class: WHO functional class; SO2: oxygen saturation, mRASP: modified risk assessment score of PAH; COMPERA: comparative, prospective registry of newly initiated therapies for pulmonary hypertension score; SPAHR: Swedish pulmonary arterial hypertension registry score; FPHR 3p: french pulmonary arterial hypertension registry score, 3 parameters; FPHR 4p: french pulmonary arterial hypertension registry score, 4 parameters.

[b]Numeric variables: Mann-Whitney U test with r effect size statistic; categorical variables: χ^2 test with Cramer V effect size statistic.

Supplementary Table S6: Characteristic of the participant clusters in the Linz/Vienna cohort. Numeric variables are presented as medians with interquartile ranges (IQR) and ranges. Categorical variables are presented as percentages and counts within the complete observation set.

**Variable**[a]	**Cluster #1**	**Cluster #2**	**Significance**[b]	**Effect size**[b]
Participants, n	35	48		
3-year mortality	5.7% (2)	15% (7)	ns (p = 0.38)	V = 0.14
5-year mortality	5.7% (2)	19% (9)	ns (p = 0.21)	V = 0.19
Overall mortality	14% (5)	31% (15)	ns (p = 0.17)	V = 0.2
Age, years	63 [IQR: 46 - 70]<br>23 - 81	71 [IQR: 64 - 75]<br>26 - 82	p = 0.0043	r = 0.34
SMWD, m	440 [IQR: 350 - 510]<br>190 - 620	320 [IQR: 180 - 370]<br>50 - 580	p < 0.001	r = 0.49
mPAP, mmHg	34 [IQR: 28 - 39]<br>18 - 57	44 [IQR: 35 - 50]<br>28 - 67	p < 0.001	r = 0.48
Sex	female: 80% (28)<br>male: 20% (7)	female: 56% (27)<br>male: 44% (21)	ns (p = 0.069)	V = 0.25
PVR, Wood	3.7 [IQR: 3 - 5]<br>1.4 - 10	6.6 [IQR: 4.9 - 9]<br>2.3 - 20	p < 0.001	r = 0.47
PCWP, mmHg	10 [IQR: 8 - 12]<br>1 - 27	12 [IQR: 8.5 - 14]<br>1 - 25	ns (p = 0.22)	r = 0.15
Anemia	5.7% (2)	25% (12)	ns (p = 0.069)	V = 0.25
log RDW, %	2.7 [IQR: 2.6 - 2.8]<br>2.5 - 2.8	2.8 [IQR: 2.7 - 2.8]<br>2.5 - 3.1	p = 0.0077	r = 0.32
Renal insufficiency	11% (4)	23% (11)	ns (p = 0.34)	V = 0.15
log FT, ng/ml	4.2 [IQR: 3 - 4.8]<br>1.9 - 5.7	4.4 [IQR: 3.7 - 5]<br>2.5 - 7.1	ns (p = 0.1)	r = 0.2
log TF-Sat, %	3.1 [IQR: 2.7 - 3.3]<br>1.6 - 4.1	2.9 [IQR: 2.4 - 3.4]<br>0.69 - 4.5	ns (p = 0.38)	r = 0.1
MCV, fl	89 [IQR: 84 - 93]<br>78 - 110	90 [IQR: 87 - 93]<br>76 - 100	ns (p = 0.49)	r = 0.077
log NT-pro-BNP, pg/ml	5.3 [IQR: 4.9 - 6.1]	7.3 [IQR: 6.7 - 7.9]	p < 0.001	r = 0.66

Variable[a]	Cluster #1	Cluster #2	Significance[b]	Effect size[b]
	3.2 - 7	4.8 - 10		
Percardial effusion	0% (0)	6.2% (3)	ns (p = 0.38)	V = 0.17
RAA, cm^2	17 [IQR: 15 - 17]<br>13 - 20	22 [IQR: 19 - 25]<br>15 - 30	p < 0.001	r = 0.69
CI, NA	2.8 [IQR: 2.5 - 3.1]<br>1.8 - 3.8	2.4 [IQR: 2.1 - 2.8]<br>1.4 - 3.6	p = 0.0086	r = 0.31
mRAP, mmHg	3 [IQR: 1 - 5]<br>0 - 16	8 [IQR: 5.8 - 9.2]<br>1 - 20	p < 0.001	r = 0.55
WHO class	I/II: 83% (29)<br>III/IV: 17% (6)	I/II: 31% (15)<br>III/IV: 69% (33)	p < 0.001	V = 0.51
SO2	≥95: 60% (21)<br><95: 40% (14)	≥95: 40% (19)<br><95: 60% (29)	ns (p = 0.15)	V = 0.2
mRASP, risk strata	low: 86% (30)<br>int: 14% (5)<br>high: 0% (0)	low: 4.2% (2)<br>int: 81% (39)<br>high: 15% (7)	p < 0.001	V = 0.83
COMPERA, risk strata	low: 83% (29)<br>int: 17% (6)<br>high: 0% (0)	low: 6.2% (3)<br>int: 90% (43)<br>high: 4.2% (2)	p < 0.001	V = 0.78
SPAHR, risk strata	low: 80% (28)<br>int: 20% (7)<br>high: 0% (0)	low: 0% (0)<br>int: 98% (47)<br>high: 2.1% (1)	p < 0.001	V = 0.84
FPHR 3p, number of risk factors	0: 31% (11)<br>1: 37% (13)<br>2: 23% (8)<br>3: 8.6% (3)	0: 0% (0)<br>1: 8.3% (4)<br>2: 35% (17)<br>3: 56% (27)	p < 0.001	V = 0.67
FPHR 4p, number of risk factors	0: 31% (11)<br>1: 40% (14)<br>2: 23% (8)<br>3: 5.7% (2)<br>4: 0% (0)	0: 0% (0)<br>1: 15% (7)<br>2: 27% (13)<br>3: 42% (20)<br>4: 17% (8)	p < 0.001	V = 0.66

[a]SMWD: six-minute walking distance; mPAP: mean pulmonary arterial pressure; PVR: pulmonary vascular resistance; PCWP: pulmonary capillary wedge pressure; RDW: red blood cell distribution width; FT: ferritin; TF-Sat: transferrin saturation; MCV: mean corpuscular volume; NT-pro-BNP: N-terminal pro-brain natriuretic peptide; RAA: right atrial area; CI: cardiac index; WHO class: WHO functional class; SO2: oxygen saturation, mRASP: modified risk assessment score of PAH; COMPERA: comparative, prospective registry of newly initiated therapies for pulmonary hypertension score; SPAHR: Swedish pulmonary arterial hypertension registry score; FPHR 3p: french pulmonary arterial

Variable[a]	Cluster #1	Cluster #2	Significance[b]	Effect size[b]
hypertension registry score, 3 parameters; FPHR 4p: french pulmonary arterial hypertension registry score, 4 parameters.				
[b]Numeric variables: Mann-Whitney U test with r effect size statistic; categorical variables: χ^2 test with Cramer V effect size statistic.				

Supplementary Figures

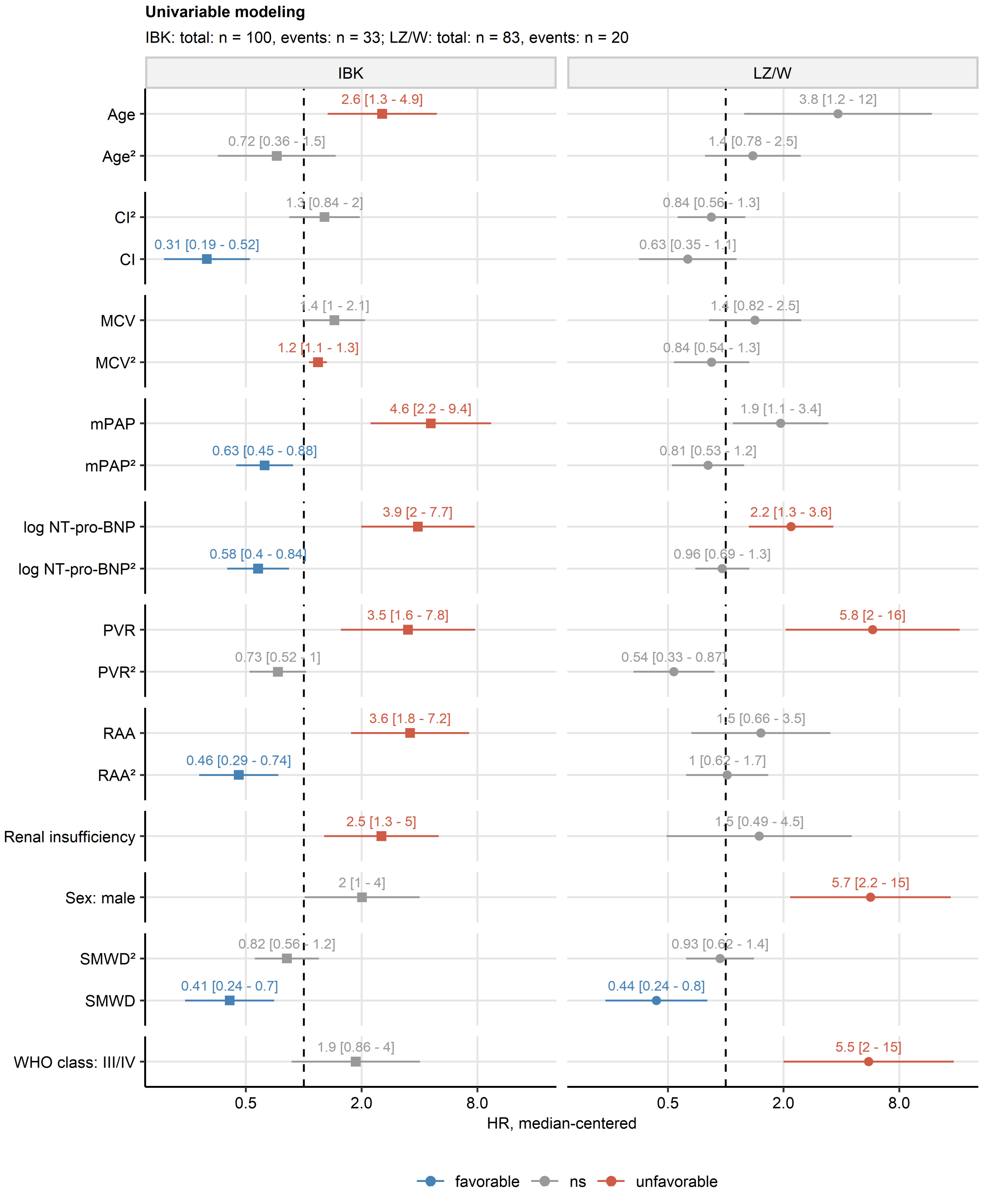

Supplementary Figure S1. Univariable Cox proportional hazard modeling.

Association of candidate risk factors (Supplementary Table S1) with overall survival was investigated with a series of univariable Cox proportional hazard models. Numeric independent variables were normalized and median-centered and their first and second order terms included in the models. Hazard ratio (HR) estimate significance was determined by Wald Z test and adjusted for multiple testing with Benjamini-Hochberg method. HR values with 95% confidence intervals for variables significantly associated with the survival in at least one Innsbruck (IBK) or Linz/Vienna cohort (LZ/W) were presented in a Forest plot. Numbers of complete observations and mortality are indicated under the plot. CI: cardiac index; MCV: mean corpuscular volume; mPAP: mean pulmonary arterial pressure; NT-pro-BNP: N terminal pro brain natriuretic peptide; PVR: pulmonary vascular resistance; RAA: right atrial area; SMWD: six minute walking distance.

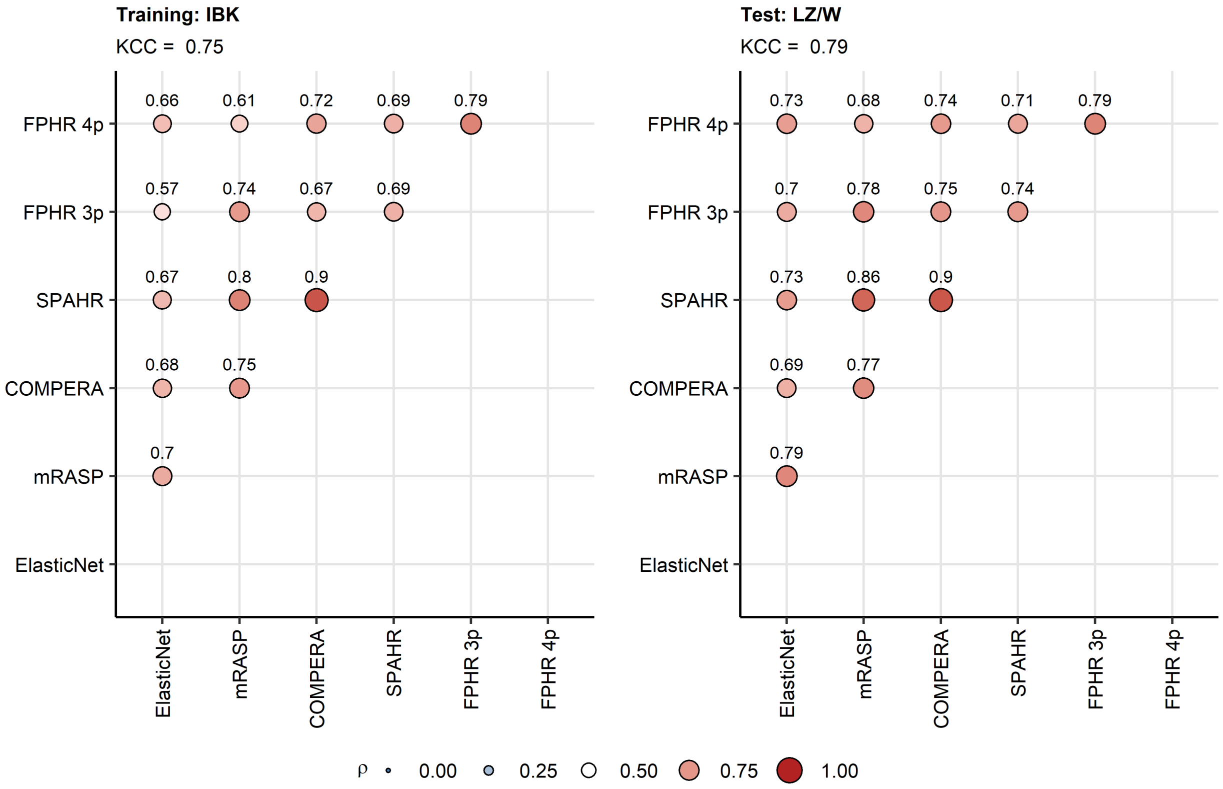

Supplementary Figure S2. Correlation of the Elastic Net signature and established risk assessment scores.

Established PAH risk scales (mRASP: modified risk assessment score of PAH; COMPERA: comparative, prospective registry of newly initiated therapies for pulmonary hypertension score; SPAHR: Swedish pulmonary arterial hypertension registry score; FPHR 3p: french pulmonary arterial hypertension registry score; FPHR 4p: french pulmonary arterial hypertension registry score, 4 parameters) were displayed in ordinal scales (mRASP, COMPERA, SPAHR: low: 1, intermediate [int]: 2, high risk: 3, FPHR scales: number of risk factors). Their correlation with the newly developed Elastic Net signature was assessed by Spearman's test corrected for multiple testing with Benjamini-Hochberg method. Consistency of the entire risk assessment battery was investigated with Kendall's coefficient of concordance (KCC). Correlation coefficients ρ are presented as points. Point size and color codes for the ρ value. Points are labeled with their ρ values. KCC values are displayed in the plot captions.

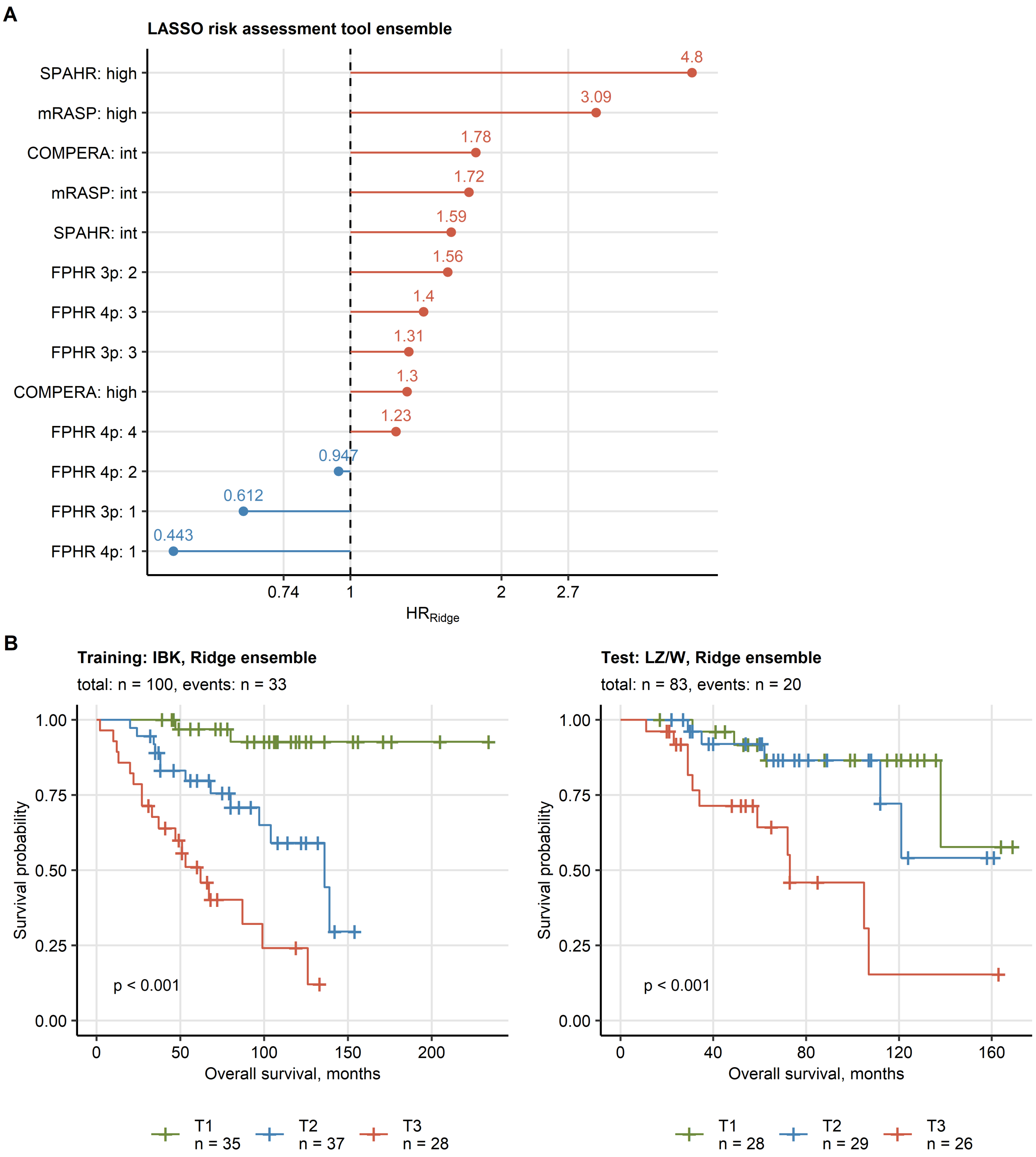

Supplementary Figure S3. Development of a Ridge ensemble model including the Elastic Net signature and established PAH risk assessment tools.

The Ridge Cox regression ensemble model was trained in the Innsbruck cohort (IBK) with the established PAH risk scales (mRASP: modified risk assessment score of PAH; COMPERA: comparative, prospective registry of newly initiated therapies for pulmonary hypertension

score; SPAHR: Swedish pulmonary arterial hypertension registry score; FPHR 3p: french pulmonary arterial hypertension registry score; FPHR 4p: french pulmonary arterial hypertension registry score, 4 parameters) as explanatory variables. The Elastic Net signature was expressed as a numeric variable, the other risk tools were included in the model as categorical variables (mRASP, COMPERA, SPAHR: low, intermediate [int], high risk: 3, FPHR scales: number of risk factors). Numbers of complete observations and mortality is indicated in B.

(A) Non-zero model coefficients represented as hazard ratios (HR). Plot points are labeled with their HR values.

(B) Association of overall survival with the Ridge ensemble linear prediction score in the training IBK and test Linz/Vienna (LZ/W) cohort was assessed by Kaplan-Meier analysis. Significance of the survival differences in the study participants stratified by the linear predictor score tertiles (T1: 0 - 33, T2: 34 - 66, T3: 66 - 100 percentile) was determined by log-rank test adjusted for multiple testing with Benjamini-Hochberg method. P values are shown in the plots, numbers of complete observations and mortality are indicated in the plot captions.

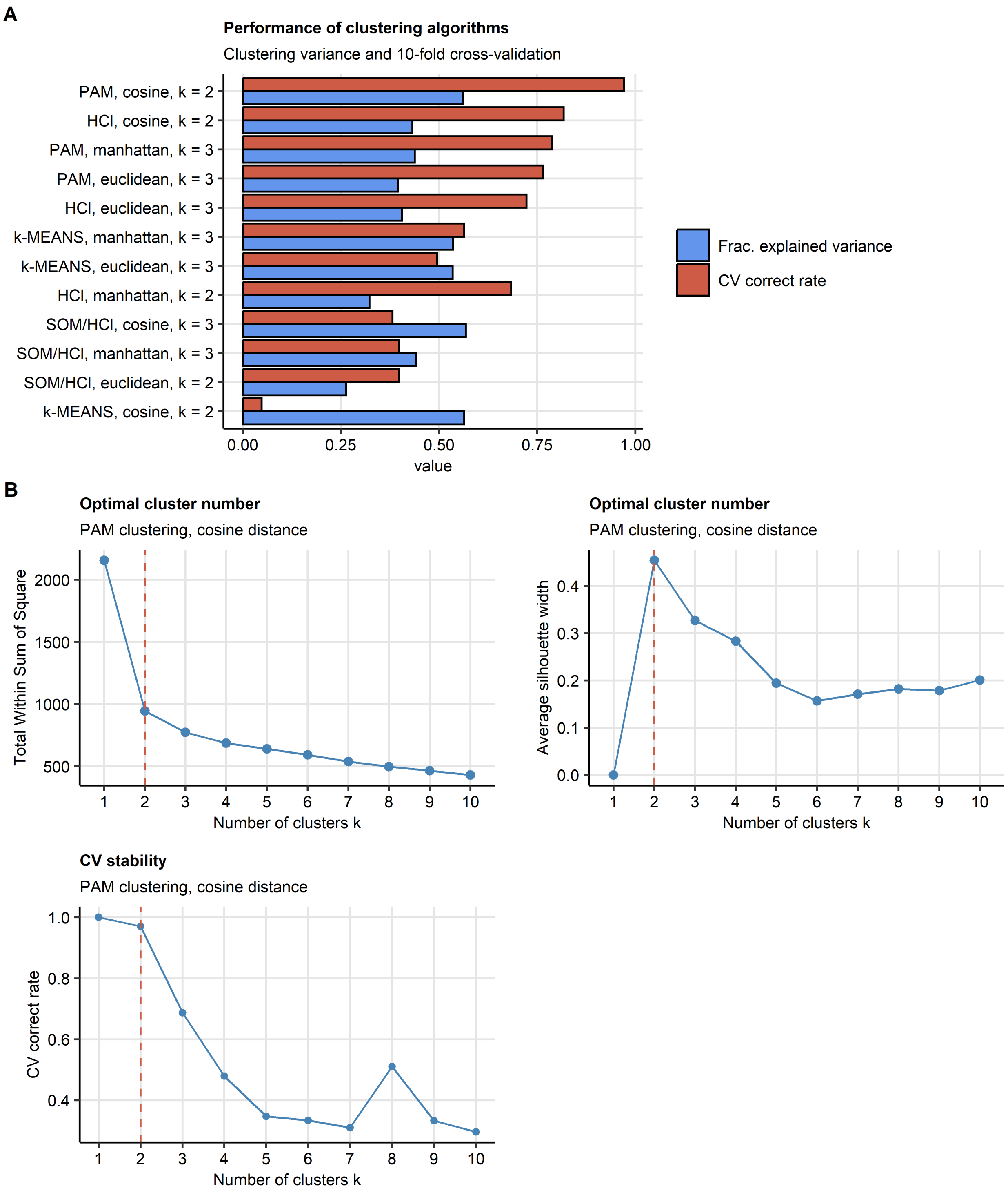

Supplementary Figure S4. Development of the PAH clusters.

Clustering of the training Innsbruck (IBK) cohort participants in respect to the survival-associated factors identified by elastic-net modeling (Figure 2A) was investigated by PAM (partitioning around medoids) algorithm and cosine distance measure.

(A) Comparison of the 'explained' clustering variance (ratio of between-cluster sum of squares to total sum of squares) and correct cluster assignment rate in 10-fold cross-validation (CV) for clustering of the IBK cohort with various algorithms (PAM, HCl: hierarchical clustering, k-means and SOM/HCl: combined self-organizing map/hierarchical clustering) and distance statistics (Euclidean, Manhattan and cosine distance) and cluster numbers k. Number of clusters k for each clustering procedure was determined by the bend of the curve of within-cluster sum of squares and maximal mean silhouette statistic. Algorithm, distance measure and the cluster number k are indicated in the Y axis. Note the superior 'explained' variance fraction and CV performance of the PAM algorithm/cosine distance procedure.

(B) Determination of the optimal cluster number for the PAM/cosine distance procedure by the bend of the total within-cluster sum of squares curve, peak of the mean silhouette statistic and correct cluster assignment rate in 10-fold CV. The dashed vertical line indicates the chosen number of PAM clusters.

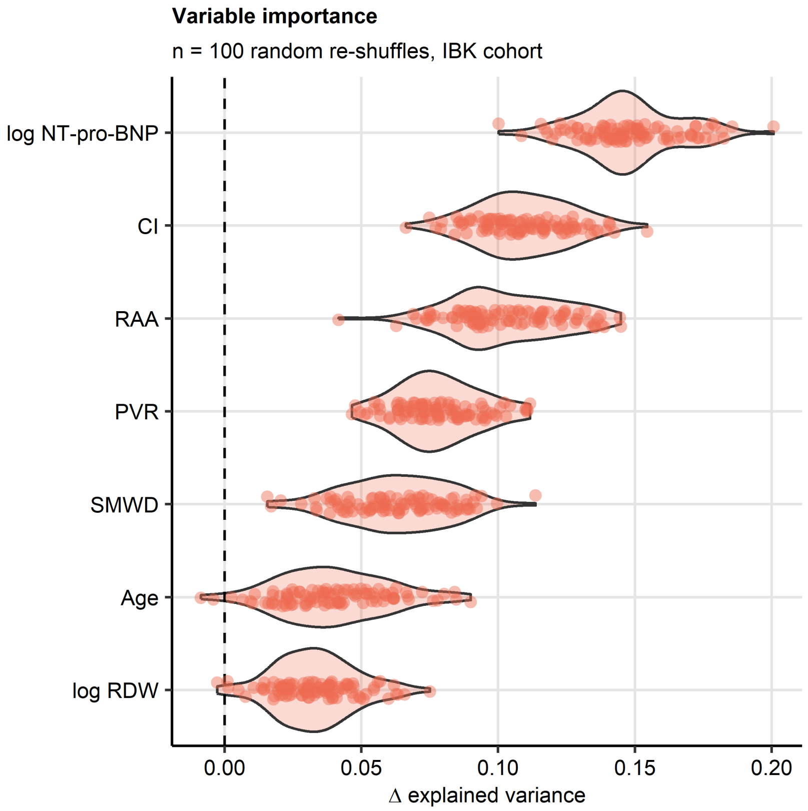

Supplementary Figure S5. Permutation importance of the variables used for development of the PAH clusters.

Importance of particular clustering factors was determined by comparing the fractions of 'explained' clustering variance (ratio of between-cluster sum of squares to total sum of squares) between the original clustering structure and clustering objects with randomly re-shuffled clustering variables in the Innsbruck (IBK) cohort. The procedure was repeated 100 times. Differences in explained clustering variances (δ) are presented in violin plots. Values for single procedure repetitions are depicted as points.

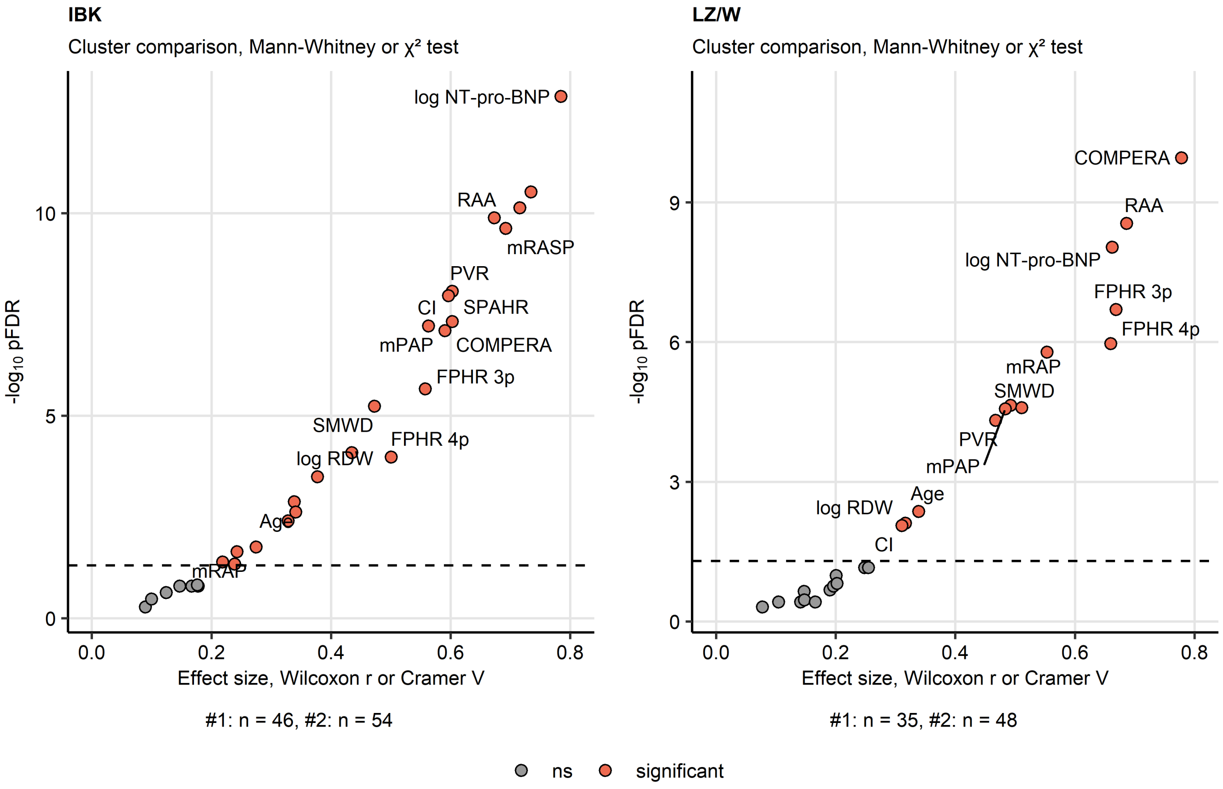

Supplementary Figure S6. Differences in study variables between the PAH clusters. *Training Innsbruck (IBK) cohort participants were clustered as presented in Figure 4 and Supplementary Figure S4. Cluster assignment of the test Linz/Vienna (LZ/W) cohort participants was accomplished by k-nearest neighbor label propagation procedure. Differences in the study variables (Supplementary Table S1) between the clusters were determined by Mann-Whitney test with r effect size statistic or by χ^2 test with Cramer V effect size statistic for numeric and categorical features, respectively. P values were adjusted for multiple testing with Benjamini-Hochberg method (pFDR). Significance (pFDR) and effect size are presented in scatter plots. Each point represents one study parameter, parameters significantly different between the clusters are highlighted in red. Parameters found significant in both cohorts are labeled with their names. The significance cutoff is depicted as a dashed line. Numbers of participants assigned to the clusters are presented under the plots. CI: cardiac index; mPAP: mean pulmonary arterial pressure; NT-pro-BNP: N terminal pro brain natriuretic peptide; PVR: pulmonary vascular resistance; RAA: right atrial area; SMWD: six minute walking distance; mPAP: mean pulmonary arterial pressure; RDW: red blood cell distribution width; mRAP: mean right atrial pressure; FPHR: French pulmonary hypertension register; SPAHR: Swedish pulmonary arterial hypertension register; COMPERA: comparative, prospective registry of newly initiated therapies for pulmonary hypertension; mRASP: modified risk assessment score of PAH.*

References

1. Wickham H, Bryan J, Posit P, Kalicinski M, Komarov V, Leitienne C, et al. readxl: Read Excel Files. 2022.

2. Wickham Hadley. ggplot2: Elegant Graphics for Data Analysis. 1st edition. New York: Springer-Verlag; 2016.

3. Henry L, Wickham Hadley. rlang: Functions for Base Types and Core R and 'Tidyverse' Features. 2022.

4. Gagolewski M, Tartanus B. Package 'stringi'. 2021.

5. Kassambara A. rstatix: Pipe-Friendly Framework for Basic Statistical Tests. 2021.

6. Signorell A. DescTools: Tools for Descriptive Statistics. 2022.

7. Therneau TM, Grambsch PM. Modeling Survival Data: Extending the Cox Model. 1st edition. New York: Springer Verlag; 2000.

8. Kassambara A, Kosinski M, Biecek P. survminer: Drawing Survival Curves using 'ggplot2'. 2016.

9. Gerds TA. pec: Prediction Error Curves for Risk Prediction Models in Survival Analysis. 2022.

10. Harrell FE. rms: Regression Modeling Strategies. 2023.

11. Friedman J, Hastie T, Tibshirani R. Regularization paths for generalized linear models via coordinate descent. Journal of Statistical Software. 2010;33:1–22.

12. Dardis C. survMisc: Miscellaneous Functions for Survival Data. 2022.

13. Kuhn M. Building predictive models in R using the caret package. Journal of Statistical Software. 2008;28:1–26.

14. Schubert E, Rousseeuw PJ. Faster k-Medoids Clustering: Improving the PAM, CLARA, and CLARANS Algorithms. In: Lecture notes in computer science (including subseries lecture notes in artificial intelligence and lecture notes in bioinformatics). Springer; 2019. p. 171–87.

15. Drost H-G. Philentropy: Information Theory and Distance Quantification with R. Journal of Open Source Software. 2018;3:765.

16. Kassambara A, Mundt F. factoextra: Extract and Visualize the Results of Multivariate Data Analyses. 2020.

17. Konopka T. umap: Uniform Manifold Approximation and Projection. 2022.

18. Gohel D. flextable: Functions for Tabular Reporting. 2022.

19. Wilke CO. Fundamentals of Data Visualization: A Primer on Making Informative and Compelling Figures. 1st edition. Sebastopol: O'Reilly Media; 2019.

20. Allaire J, Xie Y, McPherson J, Luraschi J, Ushey K, Atkins A, et al. rmarkdown: Dynamic Documents for R. 2022.

21. Xie Y. knitr: A General-Purpose Package for Dynamic Report Generation in R. 2022.

22. Xie Y. Bookdown: Authoring books and technical documents with R Markdown. 2016.

23. Harrington DP, Fleming TR. A Class of Rank Test Procedures for Censored Survival Data. Biometrika. 1982;69:553.

24. Benjamini Y, Hochberg Y. Controlling the False Discovery Rate: A Practical and Powerful Approach to Multiple Testing. Journal of the Royal Statistical Society: Series B (Methodological). 1995;57:289–300.

25. Grambsch PM, Therneau TM. Proportional Hazards Tests and Diagnostics Based on Weighted Residuals. Biometrika. 1994;81:515.

26. Harrell FE, Lee KL, Mark DB. Multivariable prognostic models: Issues in developing models, evaluating assumptions and adequacy, and measuring and reducing errors. Statistics in Medicine. 1996;15:361–87.

27. Graf E, Schmoor C, Sauerbrei W, Schumacher M. Assessment and comparison of prognostic classification schemes for survival data. Statistics in Medicine. 1999;18:2529–45.

28. Simon N, Friedman J, Hastie T, Tibshirani R. Regularization Paths for Cox's Proportional Hazards Model via Coordinate Descent. Journal of Statistical Software. 2011;39:1–3.

29. Royston P, Altman DG. External validation of a Cox prognostic model: Principles and methods. BMC Medical Research Methodology. 2013;13:33.

30. Lange T, Roth V, Braun ML, Buhmann JM. Stability-based validation of clustering solutions. Neural Computation. 2004;16:1299–323.

31. Rousseeuw PJ. Silhouettes: A graphical aid to the interpretation and validation of cluster analysis. Journal of Computational and Applied Mathematics. 1987;20 C:53–65.

32. Sonnweber T, Tymoszuk P, Sahanic S, Boehm A, Pizzini A, Luger A, et al. Investigating phenotypes of pulmonary COVID-19 recovery: A longitudinal observational prospective multicenter trial. eLife. 2022;11.

33. Leng M, Wang J, Cheng J, Zhou H, Chen X. Adaptive semi-supervised clustering algorithm with label propagation. Journal of Software Engineering. 2014;8:14–22.

34. Sahanic S, Tymoszuk P, Ausserhofer D, Rass V, Pizzini A, Nordmeyer G, et al. Phenotyping of Acute and Persistent Coronavirus Disease 2019 Features in the Outpatient Setting: Exploratory Analysis of an International Cross-sectional Online Survey. Clinical infectious diseases : an official publication of the Infectious Diseases Society of America. 2022;75:e418–31.

35. McInnes L, Healy J, Melville J. UMAP: Uniform Manifold Approximation and Projection for Dimension Reduction. 2018.
